# Four model variants within a continuous forensic DNA mixture interpretation framework: Effects on evidential inference and reporting

**DOI:** 10.1371/journal.pone.0207599

**Published:** 2018-11-20

**Authors:** Harish Swaminathan, Muhammad O. Qureshi, Catherine M. Grgicak, Ken Duffy, Desmond S. Lun

**Affiliations:** 1 Department of Anatomy & Neurobiology, Boston University School of Medicine, Boston, Massachusetts, United States of America; 2 Department of Computer Science, Rutgers University, Camden, New Jersey, United States of America; 3 Department of Chemistry, Rutgers University, Camden, New Jersey, United States of America; 4 Center for Computational and Integrative Biology, Rutgers University, Camden, New Jersey, United States of America; 5 Hamilton Institute, Maynooth University, Kildare, Ireland; 6 Department of Plant Biology, Rutgers University, New Brunswick, New Jersey, United States of America; Chuo University, JAPAN

## Abstract

Continuous mixture interpretation methods that employ probabilistic genotyping to compute the Likelihood Ratio (LR) utilize more information than threshold-based systems. The continuous interpretation schemes described in the literature, however, do not all use the same underlying probabilistic model and standards outlining which probabilistic models may or may not be implemented into casework do not exist; thus, it is the individual forensic laboratory or expert that decides which model and corresponding software program to implement. For countries, such as the United States, with an adversarial legal system, one can envision a scenario where two probabilistic models are used to present the weight of evidence, and two LRs are presented by two experts. Conversely, if no independent review of the evidence is requested, one expert using one model may present one LR as there is no standard or guideline requiring the uncertainty in the LR estimate be presented. The choice of model determines the underlying probability calculation, and changes to it can result in non-negligible differences in the reported LR or corresponding verbal categorization presented to the trier-of-fact. In this paper, we study the impact of model differences on the LR and on the corresponding verbal expression computed using four variants of a continuous mixture interpretation method. The four models were tested five times each on 101, 1-, 2- and 3-person experimental samples with known contributors. For each sample, LRs were computed using the known contributor as the person of interest. In all four models, intra-model variability increased with an increase in the number of contributors and with a decrease in the contributor’s template mass. Inter-model variability in the associated verbal expression of the LR was observed in 32 of the 195 LRs used for comparison. Moreover, in 11 of these profiles there was a change from LR > 1 to LR < 1. These results indicate that modifications to existing continuous models do have the potential to significantly impact the final statistic, justifying the continuation of broad-based, large-scale, independent studies to quantify the limits of reliability and variability of existing forensically relevant systems.

## Introduction

Within the forensic sciences, the accepted method by which to report the weight of DNA evidence in the courtroom is by presenting Likelihood Ratio (LR), which compares the probability of observing the evidence under two alternative hypotheses [1], and is expressed as:

$$LR=\frac{\mathrm{Pr}\left(E|H_{p},I\right)}{\mathrm{Pr}\left(E|H_{d},I\right)},$$

where *E* is the evidence and *H*_*p*_ and *H*_*d*_ are two competing hypotheses, and *I* is the case or contextual information. The numerator is the probability of observing the evidence given the person of interest is a contributor to the item of evidence (the prosecution’s hypothesis, *H*_*p*_) and the denominator is the probability of observing the evidence given the person of interest did not contribute to the item of evidence (the defense’s hypothesis, *H*_*d*_). The evidence shows support for the prosecution’s hypotheses if LR > 1, while if LR < 1 the defense’s hypothesis is supported [1].

Use of the LR has been recommended over other schemes such as the Random Man Not Excluded (RMNE) [1], and several continuous interpretation methods [2–6] to compute the LR have been developed and have gained currency in recent years. Continuous LR approaches, unlike binary [7] and semi-continuous methods [8, 9], evaluate most of the quantitative information in the signal. Quantitative probabilistic genotyping methods have been shown to be more robust to small quantities of DNA and have, in general, a greater ability to distinguish donors from non-donors [10, 11].

The evidence from a DNA sample is in the form of an electropherogram (epg) composed of signal from DNA fragments, baseline noise and artifacts such as stutter [12–14]. Continuous interpretation methods use probabilistic models of these processes to assign likelihoods to observed peak heights in their calculation of the LRs. Since the foundational work on forensic probabilistic genotyping was published [15], continued development of this field has resulted in numerous forensically relevant computational systems [2, 4, 5]. This work is not a comprehensive review of continuous probabilistic methods employed for human identification or a review of forensic DNA mixture interpretation, and readers are referred to [16, 17] for additional information, we discuss some differences in the models implemented in some of the more mature probabilistic genotyping systems.

All continuous methods must include an assumption about the distribution of the allele signal peak heights in the epg. For example, while Puch-Solis et al. [5] and Cowell et al. [4] use a gamma distribution to model allele peak heights, Perlin et al. [2] and Taylor et al. [3] use a normal distribution to model peak heights and the log of the ratio of observed to expected peak heights, respectively.

Moreover, not all of these methods incorporate models for noise and other non-allele signal artifacts in their calculation, but if they do they quantitatively differ in the way they account for their contribution to measured fluorescence. For example, the authors of [5] do not account for either the possibility of ‘drop in’ or for a contribution from noise, while the authors of [2–4] incorporate either drop in or noise in their models, but use distinct assumptions: Cowell et al. [4] account for drop in by adding unknown contributors with low template masses and in turn high dropout rates; Taylor et al. [3] employ a model in which drop in events either have a fixed probability of occurring or have a probability that is a function of the height of the observed peak; and Perlin et al. [2] model background noise using a normal distribution.

Stutter is a PCR amplification artifact caused by ‘strand slippage’ that generates non-allelic peaks in the signal that can be hard to distinguish from allelic peaks from minor contributors to a DNA mixture [18]. The models utilized by Puch-Solis et al. [5], Cowell et al. [4] and Perlin et al. [2] to encapsulate stutter differ from each other and account for reverse stutter (stutter that is one repeat unit shorter than the allele), while the model in [19] also incorporates the possibility of forward stutter (stutter that is one repeat unit larger than the allele).

Differences also exist in the way the models treat the underlying mixture ratio of the evidence sample, which specifies the proportions in which the contributors gave rise to the mixture and is unknown in case-work samples. Some authors assume that the mixture ratio is the same at all loci [4, 5] whereas others allow the mixture ratio to be different at distinct loci [2, 3].

Recommendations from the DNA Commission of the International Society of Forensic Genetics describing general methods that can be utilized to compute an LR that takes into account probabilities of dropout and stutter have also been published [1, 20]; however, there is no consensus regarding a standard continuous model, or whether applying the same standard model is recommended for all cases within the criminal justice system. Since the choice of model impacts the probability calculation, it is possible that changes to the underlying model will result in differences in the LR, which, in turn, may affect any verbal classification that is drawn from the evidence, if used by the forensic expert. Like the corresponding underlying model assumptions, there is also no consensus regarding methods by which forensic experts are to communicate LR results to the trier-of-fact. In an attempt to communicate the weight of the evidence to a non-scientific trier-of-fact, the adoption of a verbal scale is sometimes employed [21], though the authors of [17] have suggested example calculations or hypothetical scenarios be presented in lieu of verbal schemes. Whatever the method, without standardization, it is is the expert witness that decides the way to verbally communicate the relevance of the LR value to the trier-of-fact. More recently the United States Department of Justice released their approved *Uniform Language for Testimony and Reports–Autosomal DNA with Probabilistic Genotyping* which states that a verbal scale may be used during testimony [22] in U.S. courts of law, though the bin sizes provided in this document differ from those of those in [21]. Moreover, pursuant to the recent PCAST recommendation of establishing the validity range, for example, in terms of the number of contributors and template DNA masses of the contributors, of probabilistic systems [23, 24], the circumstances in which a system yields unreliable or differing results are of importance, especially since modifications to a model can affect the validity range of the system.

Previous studies have shown that the LR can be sensitive to assumptions regarding the number of contributors and the probability of dropout and drop in [25–27]. It has also been demonstrated that factors such as PCR and the content of allele frequency databases have an impact on the variation in the LR computed using a continuous method [28, 29]. Recent work has demonstrated that differences in output between semi-continuous and continuous systems result in clear differences in the LRs for some samples [10, 30, 31]. Despite these studies, comparisons between continuous probabilistic systems are not readily available in the literature, though some examples using small datasets do exist. For example, Morimoto et al. [31] compare the continuous system Kongoh to another continuous system, EuroForMix, and demonstrate that for most high-template simple mixtures tested the LR outcomes were similar; however differences in LRs obtained from each model were obtained for more complex mixtures wherein the authors attributed the variation in outputs as “differences in the computational principle of estimating peak height variances”. Though reports of inter-model comparisons in the scientific literature do exist, sometimes resulting in the use of multiple softwares to test one item of evidence [30], the published work use limited datasets or do not replicate the runs; thus, in this work, and pursuant to PCAST’s recommendation to publish large-scale studies, we supplement the forensic and scientific literary record by examining the variability between results obtained from four variants of CEESIt [32], a tool that computes a continuous LR for a person of interest. In addition to the LR, CEESIt computes the LR distribution by random sampling of genotypes conditioned on the defense’s hypothesis, as well as the so-called *p-*value for the LR, which is the proportion of LRs sampled that are at least as large as the LR for the person of interest.

## Materials and methods

### Calibration set

Continuous methods use the height of fluorescence peaks in the signal in their probability calculation. Characterization of the peak heights was accomplished by using single source calibration profiles with known genotypes obtained from samples amplified from a wide range of input DNA masses. For a detailed description of the method by which the calibration samples (see S1 Table for details) were created, we refer to [33]. Briefly, DNA was extracted from 27 individuals. Absolute DNA quantification was performed using real-time PCR and the Quantifiler Duo Quantification kit according to the manufacturer’s recommended protocol and one external calibration curve [34]. The extracted DNA was amplified using the manufacturer’s recommended protocol for AmpFℓSTR Identifiler Plus Amplification Kit (Life Technologies, Inc.) [35]. Separation of the STR fragments was accomplished with a 3130 Genetic Analyzer using an injection voltage of 3 kV and an injection time of 10 seconds. Analysis was performed using GeneMapper ID-X v1.1.1 (Life Technologies, Inc.) and an RFU threshold of 1. A threshold of 1 RFU was used in order to capture all peak height information, i.e. the allelic, noise, and stutter peaks, in the signal. Known artifacts such as pull-up, spikes, -A, and artifacts due to dye dissociation were manually removed, as detailed in [33].

### Testing set

A total of 101, 1-, 2- and 3-person samples were used to test the four models in this study (see S2 Table for details). These 1-person test samples were created using the same protocol described for the single source samples in the calibration set. Multi-person samples were created by mixing appropriate volumes of the single source DNA extracts to attain the various ratios specified in S3 Table. Once mixed, these samples were re-quantified and then amplified using the target masses from S2 Table. The 1-person samples contained DNA from 30 different individuals, the 2-person samples contained DNA from 6 different individuals (3 combinations) and the 3-person samples contained DNA from 6 different individuals (2 combinations). None of the contributors to the calibration set were present in the testing set and none of the contributors to the testing set were present in the calibration set.

### Models and allele frequencies

The four probabilistic models, called A, B, C, and D, used in this study employ the assumption that the allele heights, noise peak, and stutter ratios are either normally or lognormally distributed. The functions used to model the variables (such as dropout rate, mean of noise peak heights, etc.) with respect to the DNA mass were chosen by fitting the calibration data with MATLAB (R2015b, The Mathworks, Natick, Massachusetts) and are shown in S4 Table. The allele frequencies used in this study were those of the US Caucasian population published in [35].

### Algorithm

For purposes of this work, we use the true number of contributors, *n*, for the analysis of each sample. We employ the following alternative hypotheses for *H*_*p*_ and *H*_*d*_ in the LR calculation.

*H*_*p*_: The evidence is a mixture of data from the suspect (with genotype *s*) and *n*−1 other unknown, not necessarily related contributors, whom we term the interference contributors.

*H*_*d*_: The evidence originates from *n* unknown individuals not necessarily related to the suspect.

The four models tested are variants of the probabilistic models used by CEESIt [32]. These models were chosen to reflect common modeling assumptions in the published literature, as discussed in the Introduction.

The original CEESIt algorithm is described in detail in [32], but since its publication, improvements to it have been made. In the following, we describe the algorithm used to generate the results of this paper.

Let *E* denote the evidence in the form of the electropherogram (epg); let *R* denote the genotype of the assumed contributor; let *N* denote the number of contributors; for *N* = *n*, let **Θ** be the vector with components Θ_*i*_ that represent the mixture proportion of each contributor *i* ∈ {1,…,*n*}, so that **Θ** takes values in $\Delta^{n-1}=\{(\Theta_{1},\ldots ,\Theta_{n}){\in R}^{n}|\sum_{i=1}^{n}\Theta_{i}=1,\Theta_{i}>0\;\forall \;i)$ the unit *n*−1 simplex; and let *f*_**Θ**_ denote the probability density function of **Θ**. For models A, C and D, this density is assumed to be uniform over Δ^*n*−1^ and that **Θ** is the same over all loci. For model B, it is assumed that the contributor mixture proportions at each locus are independent and identically distributed as uniform distributions over Δ^*n*−1^.

For all models apart from B, to calculate the numerator of the LR, we first integrate over the sample space:

$$\mathrm{Pr}\left(E|R=s,N=n\right)=\underset{\theta \in \Delta^{n-1}}{\int }\mathrm{Pr}\left(E|\Theta =\theta ,R=s,N=n\right)f_{\Theta }\left(\theta \right)d\theta ,$$

This integral is approximated using a fixed set of mixture ratios in Δ^*n*−1^ for each *n*. This set of mixture ratios was determined by employing k-means clustering [32] to uniformly distribute the set of ratios over the simplex and are specified in S3 Table.

Let ***L*** be the set of all loci in the evidence sample, *E*_*l*_ be the evidence at locus *l* and *s*_*l*_ be the genotype of the suspect at locus *l*. The STR loci used for forensic DNA analysis are assumed to be in linkage equilibrium and independent of each other, conditioned on the mixture ratio [36]. Hence, we obtain:

$$\mathrm{Pr}\left(E|\Theta =\theta ,R=s,N=n\right)=\prod_{l\in L}\mathrm{Pr}\left(E_{l}|\Theta =\theta ,R_{l}=s_{l},N=n\right).$$

For Model B, which assumes that the mixture ratio is independent across loci, the probability of observing the evidence is calculated by taking the product of the probability of observing the evidence at all the loci, which in turn is computed by integrating over the sample space of the mixture ratios:

$$\mathrm{Pr}\left(E|R=s,N=n\right)=\prod_{l\in L}\underset{\theta \in \Delta^{n-1}}{\int }\mathrm{Pr}\left(E_{l}|\Theta =\theta ,R=s,N=n\right)f_{\Theta }\left(\theta \right)d\theta$$

The probability of observing the evidence at a locus *l* is calculated by using importance sampling on the genotypes of the interference contributors:

$$\mathrm{Pr}\left(E_{l}|\Theta =\theta ,R_{l}=s_{l},N=n\right)\approx \frac{\sum_{i=1}^{J}Pr(E_{l}|U_{i}^{n-1}=u_{i}^{n-1},\Theta =\theta ,{R_{l}=s}_{l},N=n)w_{i}}{J},$$

where *J* is the number of interference samples; $U_{i}^{n-1}=(U_{i}^{1},\ldots ,U_{i}^{n-1})$ is a vector of the random genotypes of *n*−1 contributors; $w_{i}=P(u_{i}^{n-1})/Q(u_{i}^{n-1})$ is the weight of sample *i*, where $P(u_{i}^{n-1})$ is the probability of the interference genotypes under the allele frequency distribution and $Q(u_{i}^{n-1})$ is the probability of the interference genotypes under the peak height distribution. The number of genotype samples *J* is not a constant in the CEESIt framework. Genotype samples are generated in batches until the probability converges such that the difference in Pr(*E*_*l*_|**Θ** = ***θ***,*R*_*l*_ = *s*_*l*_,*N* = *n*) is less 1%.

Since the publication of [32], we have updated the model used by CEESIt for calculating the probability of observing the peak heights given the genotypes of the contributors and the mixture proportion. See the S1 Appendix for a description of the computation of Pr(*E*_*l*_|***G*** = ***g***,**Θ** = ***θ***,*N* = *n*), which is the probability of observing the evidence (peak heights) at a locus *l*, given the genotypes of the contributors ***g***, the mixture proportions ***θ*** and the number of contributors *n*. The value of Pr(*E*_*l*_|***G*** = ***g***,**Θ** = ***θ***,*N* = *n*) depends on models of peak height distributions for peaks arising from alleles, stutter, and noise, which are derived from the calibration set. To calculate this quantity, we did not use an analytical threshold to filter out peaks below the threshold, which is a common practice in operational settings. We chose not to apply an analytical threshold because provided the model of the signal, stutter, and noise is reasonable, the true value of Pr(*E*_*l*_|***G*** = ***g***,**Θ** = ***θ***,*N* = *n*) is only obscured, not improved, by applying an analytical threshold. In particular, because many of the samples used for this study are low-template samples, an analytical threshold could potentially filter out a significant number of allelic peaks. Thus, rather than applying an analytical threshold, we focused on developing and utilizing models that describe the signal, stutter, and noise reasonably well.

Let ***R***_**1**_ be a set consisting of all genotypes *r* such that {Pr(*E*_*l*_|*R*_*l*_ = *r*_*l*_) ≄ 0 for all loci *l*}, where “≃0” means “evaluates to 0 using double-precision 64-bit floating-point arithmetic”. To calculate an approximation of the LR and the *p*-value of the LR, CEESIt samples 1 billion (10^9^) genotypes *r*^*i*^ from the set ***R***_**1**_\{*s*}. The LR is calculated as follows:

$$\frac{\mathrm{Pr}\left(E|H_{p}\right)}{\mathrm{Pr}\left(E|H_{d}\right)}=\frac{\mathrm{Pr}\left(E|R=s,N=n\right)}{\mathrm{Pr}\left(E|N=n\right)}\approx \frac{Pr(E|R=s,N=n)}{\mathrm{Pr}\left(E|R=s,N=n\right)\mathrm{Pr}\left(R=s\right)+Pr(R\in R_{1}/\{s\})\sum_{i=1}^{M}\mathrm{Pr}\left(E|R=r^{i},N=n\right)/M}$$

where *M* = 10^9^ [37].

The *p-*value of the LR is calculated as:

$$p‑\mathrm{value}(s)=\mathrm{Pr}(R=s)+\mathrm{Pr}\left({R\in R}_{1}\backslash \{s\}\right)\frac{\sum_{i=1}^{M}1\left(\left(Pr(E|R=r^{i})\geq Pr(E|R=s\right)\right)}{M}.$$

### Study design

The objective of this study is to investigate the stability of LRs over multiple probabilistic genotyping systems that employ similar, but not the same, model assumptions using the computational framework of CEESIt. Table 1 summarizes the different modeling assumptions of the four models. In these, we change assumptions on the mixture ratio, the underlying distribution of noise peak heights and the consideration of forward stutter peaks, each of which alters Pr(*E*|*R* = *s*,*N* = *n*). In its own right, each model is arguably “reasonable” and resembles a model structure from the literature.

**Table 1:** The four continuous models tested in this study and their modeling assumptions.

Parameter	Model A	Model B	Model C	Model D
Mixture ratio	Constant across loci	Can vary across loci	Constant across loci	Constant across loci
Noise peak height distribution	Normal	Normal	Lognormal	Lognormal
Forward stutter	Included	Included	Included	Not included

#### Model A

In this model, the mixture ratio is assumed to be constant across all the loci and integrated over their sample space, consistent with the assumptions in [4] and [5]. Noise peak heights are modeled using a normal distribution and forward stutter peaks are included in the calculation. We note that differences between this Model A and the original algorithm published in [32] exist; that is, the computation of Pr(*E*_*l*_|***G*** = ***g***,**Θ** = ***θ***,*N* = *n*) was updated to be more precise (see S1 Appendix).

#### Model B

This model is similar to Model A in all but one aspect–the underlying mixture ratio of the sample is modified. The mixture ratio specifies the proportion of a sample contributed by each individual (e.g., the major and minor contributors in a mixture, if any) and is unknown for an evidence sample. The mixture ratio can be treated in the probability calculation in at least two ways: a) assuming that the mixture ratio is constant across all the loci and integrating over the sample space of values that the mixture ratio can take or b) allowing the individual locus mixture ratios to be independent of each other. To study the impact of changing this assumption, we developed Model B, which does not assume that the mixture ratio is the same at all the markers but instead assumes that the mixture ratio varies independently from one locus to another.

#### Model C

In this model, the distribution used to assign probabilities to noise peak heights is modified. Baseline noise peaks are frequently observed in the signal at small RFUs and can interfere with allelic peaks in samples with low template masses analyzed without an analytical threshold. As mentioned previously, published models differ in the way they describe baseline noise. Baseline noise peaks are distinct from drop-in peaks, which arise from small fragments of DNA that are present during amplification and are amplified along with the DNA found within the sample. Drop-in is not incorporated in CEESIt’s model but is accounted for in the models described in [3] and [4]. In the model in [32], a normal distribution was used to describe noise peak heights. However, a recent study from this lab [13] suggested that a lognormal distribution provides a better description than the normal distribution for the noise peak heights. Hence, in Model C we use a lognormal distribution instead of a normal distribution to model the noise peak heights.

#### Model D

In this model, the possibility of forward stutter is removed from the model. Stutter peaks are observed frequently and have heights that are positively correlated to the height of the allelic peak. They can, in particular, cause problems when dealing with low template samples as stutter peak heights can be similar to minor contributors’ peak heights. While reverse stutter or *n*−1 stutter is the most common type of stutter, forward stutter or *n*+1 stutter can also occur [18]. The models published in [2, 4, 5] account for reverse stutter and do not account for the possibility of forward stutter, while [19] incorporates forward stutter into its modeling framework. Even though all these models account for stutter, they differ in the way they quantify the likelihood of its occurrence and fluorescence contribution. For example, the authors of [2, 3] model the expected stutter peak height as being linearly proportional to the allele peak height, while the authors of [5] model stutter peak heights using a gamma distribution in which the height of the peak depends upon the total peak height at the locus and the size of the parent allele giving rise to stutter; and [4] describes a model of stutter heights using a gamma distribution as a function of the mixture proportion. To study the impact of incorporating forward stutter on the LR value, in Model D we ignore the occurrence of forward stutter and instead treat a peak in the forward stutter position of an allelic peak as a noise peak.

## Results

### Small LRs stem from low template masses and small *p*-values from large LRs

The four models used in this study were tested on all the true contributors to the samples in the testing set. Thus, a 1-person sample resulted in one LR, a 2-person sample resulted in two LRs and a 3-person sample resulted in three LRs. Since a sampling algorithm was used to calculate the numerator (sampling of the genotypes of unknown contributors in mixtures) and the denominator (sampling of the genotypes of random contributors), the LR value varies from run to run. To analyze the run-to-run variation of the four models in this study, each model was run five times on all the samples in the testing set.

The *p*-value for a person of interest with genotype *s* and corresponding likelihood ratio *LR*(*s*) is defined as

$$p‑\mathrm{value}(s)=Pr(LR\geq LR(s)|H_{d}),$$

i.e. the *p*-value is the probability that a person chosen at random from the population has an LR greater than or equal to the person of interest’s LR. The *p*-value computed by the method described under ‘Algorithm’ is not exact–it is an estimate of the *p-*value calculated by randomly sampling a large number of genotypes, where we used 1 billion (10^9^), from the population. In cases where no genotype *g* with a Pr(*E*|*G* = *g*,*N* = *n*) greater than that of the person of interest was sampled, only an upper limit to the *p*-value is reported. Hence when displaying the results, 10^-9^ was used as an upper bound on the *p*-value since 10^9^ random genotypes were sampled.

A summary of the LRs and *p*-values from the four models after five runs on each sample is provided in Table 2. In addition, a comparison of the mean LR from five runs computed by each pair of models is shown in S1 Fig and S2 Fig. Each model produced 150 LRs (30 samples × 1 contributor/sample × 5 runs) for the 1-person samples, 410 LRs (41 samples × 2 contributors/sample × 5 runs) for the 2-person samples, and 450 LRs (30 samples × 3 contributors/sample × 5 runs) for the 3-person samples, giving a total of 1010 LRs. The majority (95.17%) of the LRs were greater than or equal to 1, correctly indicating support for the prosecution’s hypothesis. In the instances where the LR was less than 1, this could be explained due to a low starting template mass from the individual and in turn high levels of dropout and stutter. The *p*-values decreased with an increase in the LR, and we calculated a Spearman’s rho of -0.75 between the two quantities (see S3 Fig). This relationship is expected since the *p-*value is upper bounded by 1/LR [38].

**Table 2:** Summary of the LRs and p-values for the true contributors to the samples in the testing set.

	Model A			Model B			Model C			Model D		
Number of contributors	1	2	3	1	2	3	1	2	3	1	2	3
Minimum LR	10^−75^	10^−2^	10^−9^	10^−74^	10^−3^	10^−12^	10^−69^	10^−1^	10^−8^	10^−2^	10^−5^	10^−15^
Maximum LR	10^31^	10^38^	10^25^	10^31^	10^30^	10^15^	10^31^	10^32^	10^19^	10^31^	10^30^	10^20^
Maximum *p*-value	< = 10^−9^	10^−3^	10^−1^	< = 10^−9^	10^−3^	10^−1^	10^−4^	10^−2^	10^−1^	10^−7^	10^−2^	10^−1^
Number of LRs < 1	10	1	30	10	8	26	5	2	30	2	6	65
Number of *p*-values > 10^−9^	0	61	177	0	68	193	5	77	195	5	90	249

Each model was run five times on all the samples. If none of the 10^9^ genotypes that are stochastically sampled had an LR greater than the suspect’s, then this is reported as ≤ 10^−9^, as given by the bound *p*-value ≤ 1/*LR*.

### Intra-model variation of the LR verbal class

Since the four models tested employ a sampling algorithm to calculate the LR, we ran each model five times on each sample to report the run-to-run variation in the LR alongside the between-run LR variations. Each model resulted in 202 sets of LRs (with each set consisting of 5 LRs from the 5 runs): 30 LRs from the 1-person samples plus 82 LRs from the 2-person samples plus 90 LRs from the 3-person samples.

Verbal expressions corresponding to the LR have been discussed as a potential way to express and compliment the LR within the field of human identification [39], with recent publications from the U.S. DOJ appropriating their use. This is a system in which the value of the LR is translated to a verbal expression indicating the degree of strength the evidence shows for one proposition when compared with the other. To analyze the impacts of the run-to-run variation in the LR on the verbal classification, the set of verbal categories associated with the LR specified by the Association of Forensic Science Providers in [39] were used (Table 3). This standard has six categories for the verbal expression ranging from ‘Weak’ for an LR between 1 and 10 to ‘Extremely Strong’ for an LR > 1 million.

**Table 3:** Standards for verbal expression of likelihood ratio (Association of Forensic Science Providers, 2009).

Numerical value	Verbal expression
1–10	Weak
10–100	Moderate
100–1,000	Moderately strong
1,000–10,000	Strong
10,000–1,000,000	Very strong
> 1,000,000	Extremely strong

As is typically the case, binning of LRs used for the verbal expression determines whether the LRs from the different runs result in the same verbal expressions potentially presented to the trier-of-fact. A ‘coarse’ binning typically leads to most LRs falling in the same bin and results in the same verbal interpretation, while a ‘fine’ binning necessarily leads to more LRs falling in different bins and results in distinct verbal interpretations. In the verbal equivalent expressions used in this study, apart from very strong, the confidence designation increases by one level for every increase of one order of magnitude in the LR. We note that categorizing a continuous estimate, such as the LR, into bins has not acquired full consensus in the scientific literature, and alternate recommendations to this scheme are, for example, presented in [17].

**Fig 1:**
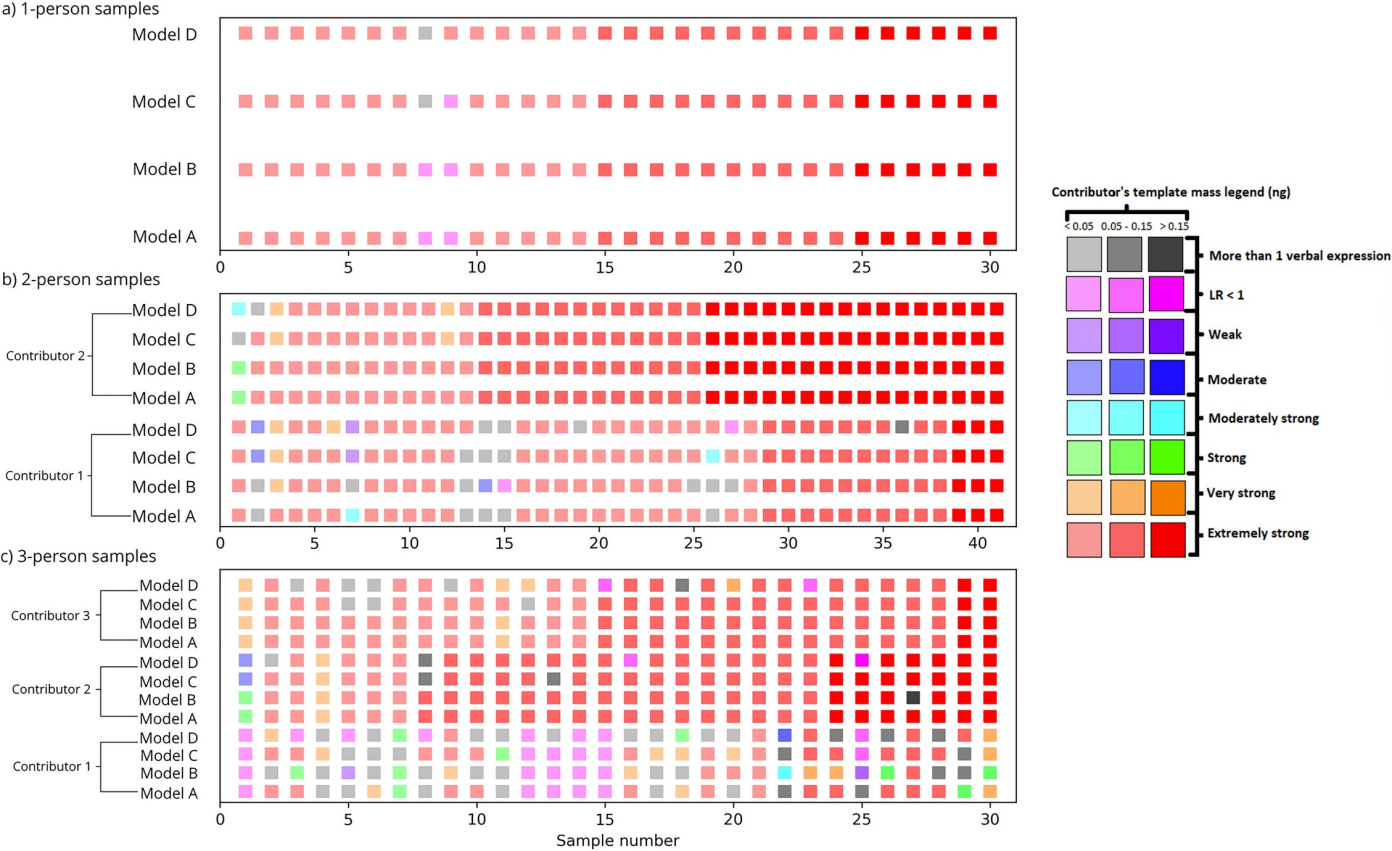
Variation of the LR within and between models. The verbal expression corresponding to the LRs from five runs for the true contributors to all the samples in the testing set is shown. For each set of samples (i.e. 1-person, 2-person, and 3-person samples), samples are numbered starting from 0 in increasing order of the total template mass. The samples that resulted in inter-model variation are as follows: sample 9 in one-person profiles; samples 1, 3, 12 (Contributor 2), 3, 6, 7, 26 and 27 (Contributor 1) in two-person profiles; samples 1, 16, 25 (Contributor 3), 11, 12, 15, 20, 23 (Contributor 2), 2, 3, 5, 8, 9, 16, 18, 20, 22, 23, 24, 25, 26, 29 and 30 (Contributor 1) in three-person profiles.

In the majority of cases–i.e., 91.34% (738 out of 808) of the cases–the LRs from all five runs fell in the same category or bin, resulting in the same verbal expression, or interpretation, based on the five LRs (Table 4, Fig 1). In all four models, the LRs for all the 1-person samples, except one sample for which Model C and Model D led to more than one verbal expression, fell in the same bin, indicating there is little ambiguity in demonstrating the level of support for one hypothesis over the other in single source samples. We observed that in certain 2- and 3-person mixtures, the LRs from different runs fell in different bins, leading to more than one verbal expression. These LRs were typically associated with individuals who were minor contributors or had low template masses. Of these, most cases involved LRs falling in adjacent bins leading to verbal expressions of ‘Very strong’ and ‘Strong’ or ‘Strong’ and ‘Moderately strong’.

**Table 4:** Intra-model variation in the LR.

Model	Same verbal expression from 5 runs	Different verbal expressions from 5 runs	Two verbal expressions more than one bin apart/More than two verbal expressions
A	189	13	3
B	185	17	2
C	187	15	2
D	177	25	5

Out of the 202 sets of LRs, the majority resulted in the same interpretation between runs. Intra-model variability increased with an increase in the number of contributors and with a decrease in the contributor’s template mass. The range in which the models exhibited intra-model variability differed between models.

**Fig 2:**
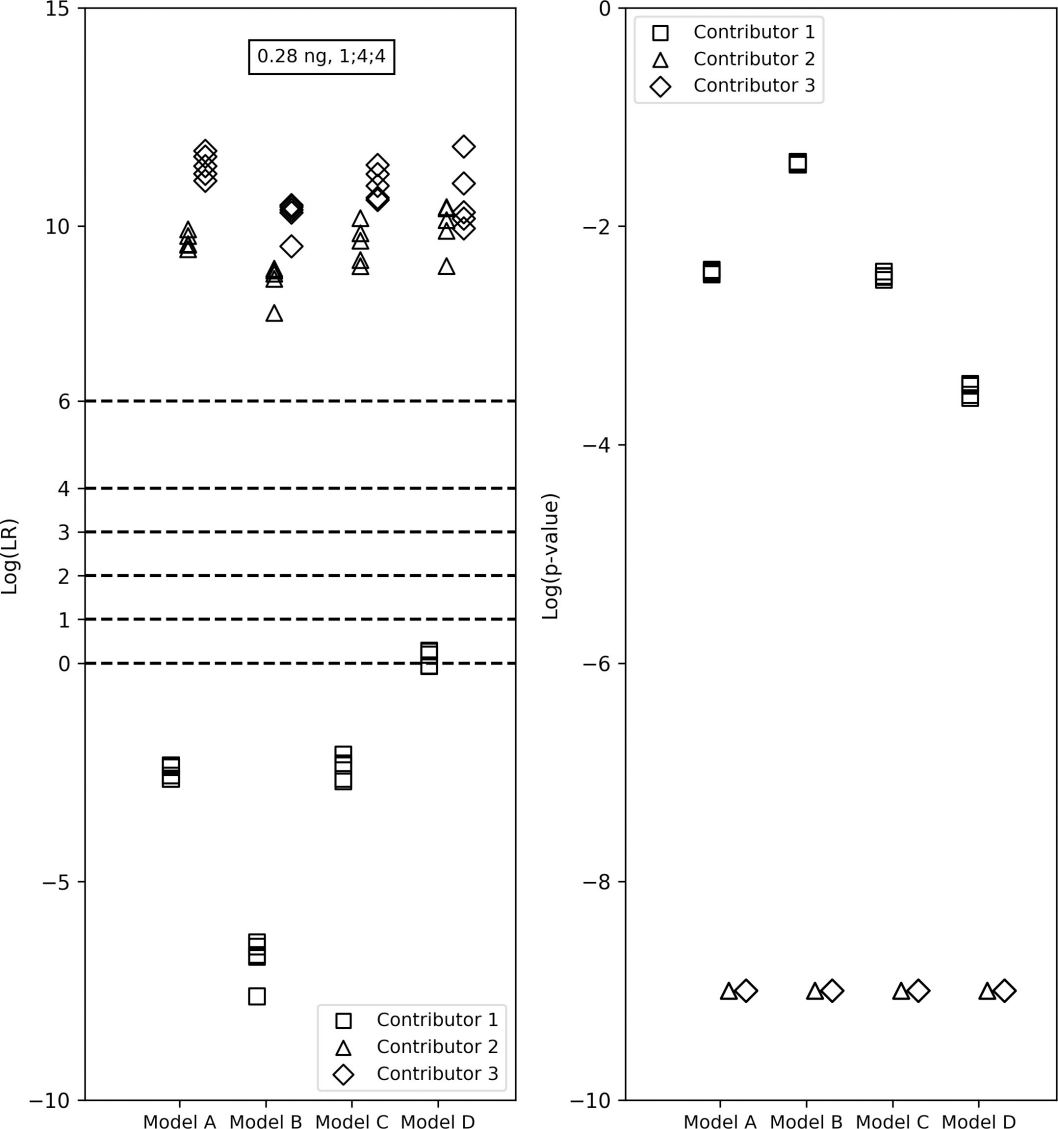
LR verbal expression levels and the LRs and *p*-values from the four models for the true contributors in a 1:4:4, 0.28ng 3-person mixture. Model D resulted in higher LRs for Contributor 1 (starting template mass: 0.03ng) than the other models—it resulted in an LR < 1 and showed ‘Weak’ support for the prosecution’s hypothesis. Models A, B and C resulted in LRs < 1 for Contributor 1. The *p*-values for Contributor 1 from all versions ranged between 10^−1^ and 10^−4^. Contributors 2 and 3 both had ‘Extremely strong’ verbal interpretations from all models and their *p*-values had an upper bound of 10^−9^.

In 12 instances, with all four models, (last column of Table 4) the LRs fell in three verbal bins or fell in two bins that were not adjacent to each other. Moreover, we observed that in one 1-person profile, two 2-person profiles and in four 3-person profiles the LRs for a contributor with a low template mass fell both above and below 1, emphasizing the uncertainty associated with evidence from contributors with low template masses. For example, in a 1:4:4 0.28ng 3-person mixture, Model D had higher LRs for Contributor 1 (starting template mass: 0.03ng) than the other models and suggested both a ‘Weak’ support for the prosecution’s hypothesis and supported the defense’s hypothesis as well (log_10_(LR)s ranging from -0.06 to 0.28). Models A, B and C resulted in LRs < 1 for Contributor 1. The *p*-values for Contributor 1 from all models ranged between 10^−1^ and 10^−4^. Contributors 2 and 3 in this sample both had ‘Extremely strong’ interpretations from all models and their *p*-values had an upper bound of 10^−9^ (Fig 2).

### Inter-model variation of the LR

Having evaluated intra-model variability, which serves as a measure of baseline variability due to the Monte Carlo design of the algorithm, we next compared the LR between the four model variants. To facilitate comparison between models, we ignore instances where there was intra-model cross-over in verbal categories and restrict our analysis to instances where two or more of the four models resulted in the same verbal expression based on the LR on all five runs (Table 5, Fig 1). In 163 of the 195 LRs used for comparison, the models compared resulted in the same verbal expression of support. In the remaining 32 LRs (from one 1-person samples, eight 2-person samples and twenty-three 3-person samples), the interpretation from one model differed from the interpretation from one or more other models. Of these 32 cases, 21 were instances where the LRs being compared were greater than 1 and resulted in verbal expressions ranging from ‘Moderate’ to ‘Extremely strong’ between the models compared.

**Table 5:** Inter-model variation in the LR.

Number of models compared	Number of LRs	Same verbal expression between models	Different verbal expressions between models
2	13	7	6
3	23	13	10
4	159	143	16
Total	195	163	32

In 163 of the 195 LRs used for comparison, the models compared resulted in the same verbal expression of support. In the remaining 32 LRs the verbal expression from one model differed from the verbal expression from one or more other models. In 11 of these 32 LRs, one or more models resulted in an LR < 1, while one or more other models showed support for the prosecution’s hypothesis. These were for contributors with low template masses.

**Fig 3:**
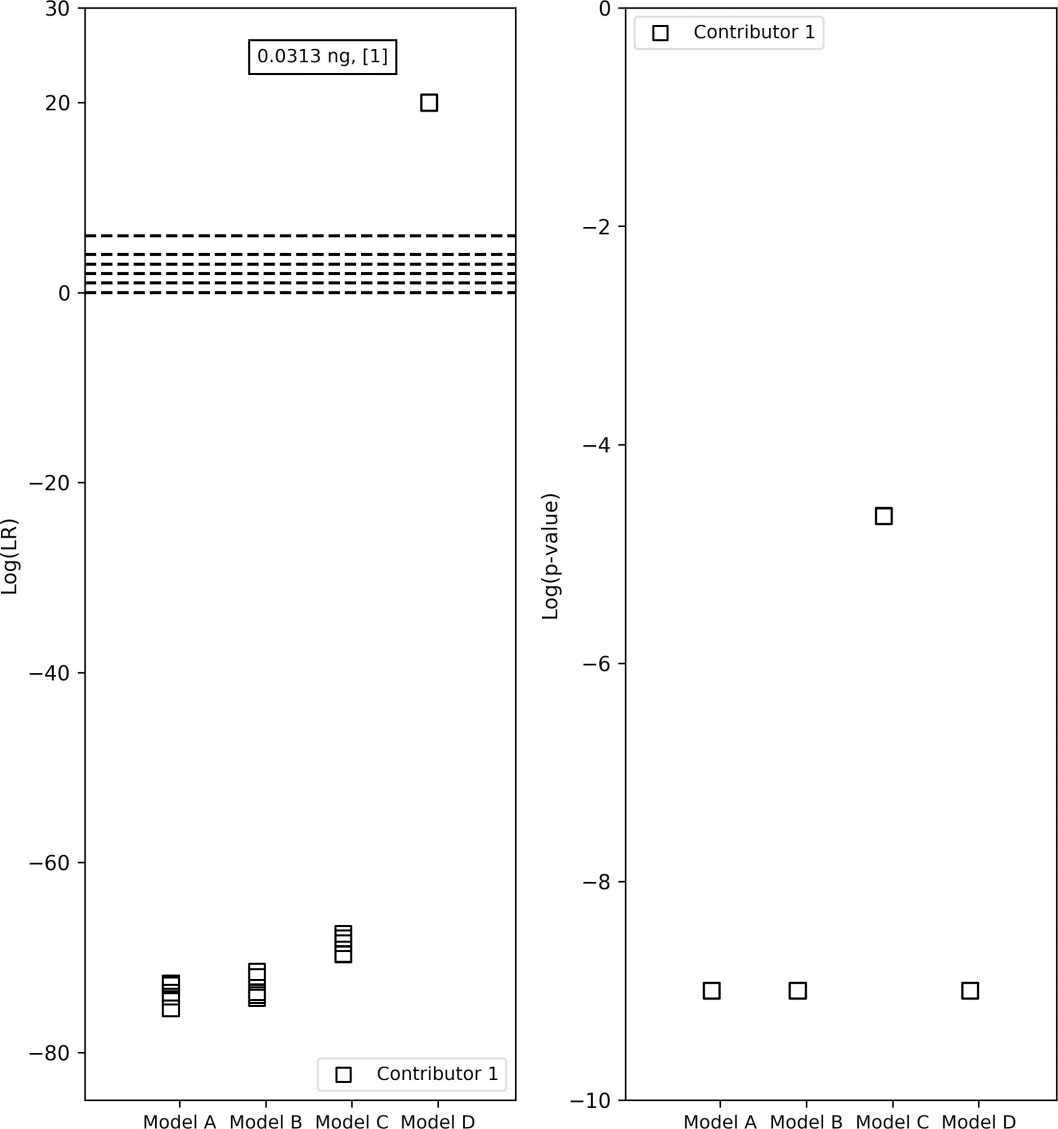
LR verbal expression levels and the LRs and *p*-values from the four models for the true contributor in a 0.03ng 1-person sample. The contributor had a LR < 1 with models A, B and C which consider forward stutter and had an LR > 1 under model D, which does not incorporate forward stutter. This occurred due to 100% forward stutter ratio at one locus, which had a low probability.

**Fig 4:**
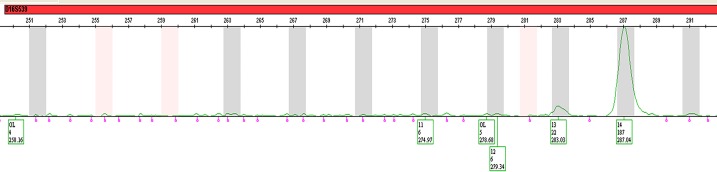
EPG of locus D16S539 in the 0.03ng 1-person sample with the LRs shown in Fig 3. Allele 11 belongs to the genotype of the contributor and has a height of 6 RFU. Allele 12 (in the forward stutter position) also has a height of 6 RFU.

**Fig 5:**
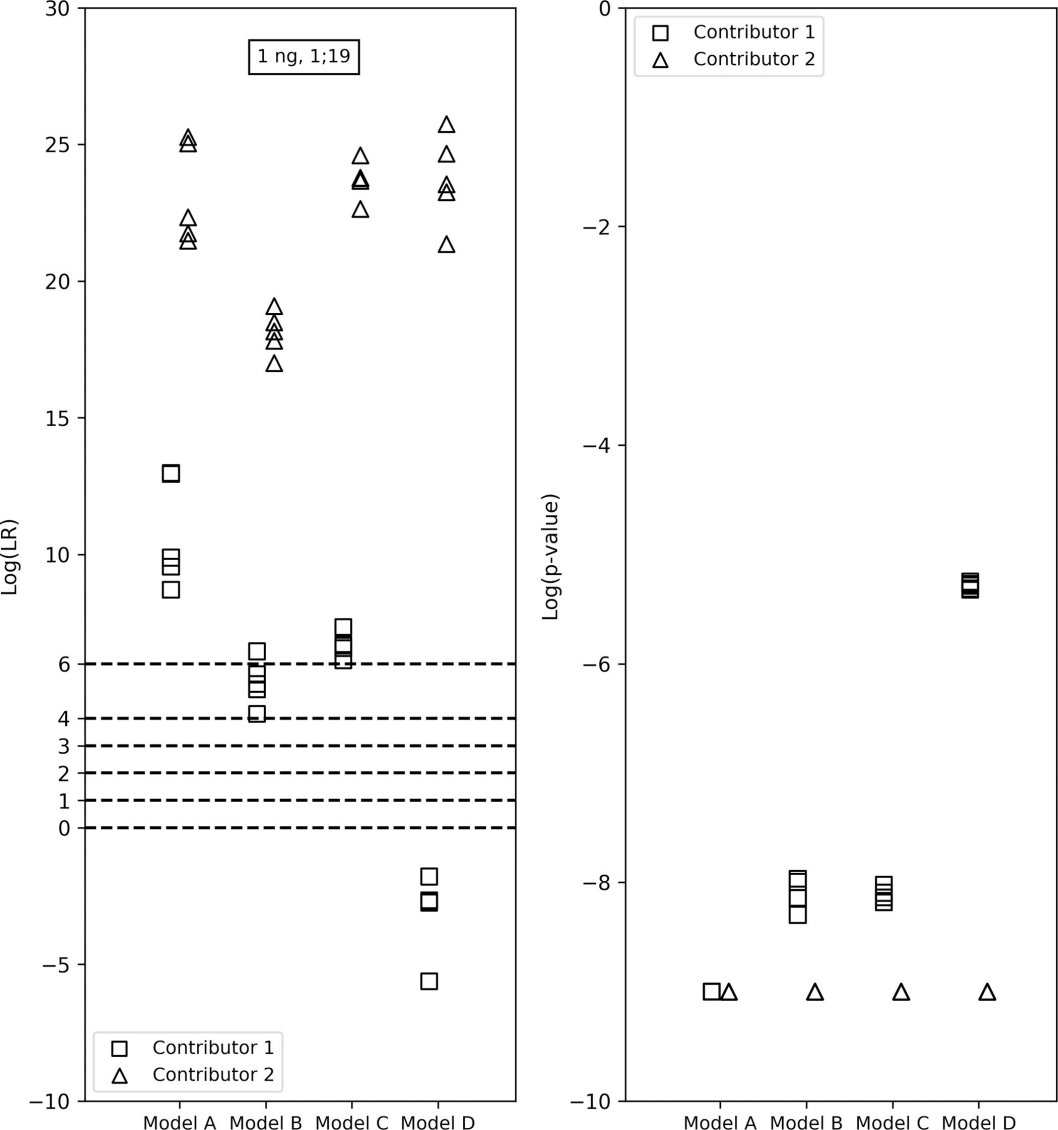
LR verbal expression levels and the LRs and *p*-values from the four models for the true contributors in a 1:19, 1ng 2-person sample. Contributor 1 had an LR < 1 under model D but had an LR > 1 under other three models because inclusion of forward stutter gave a better explanation for the heights of the peaks at reverse and forward stutter position at two loci, since reverse stutter alone was not sufficient.

Further, we observed that in the other 11 of the 32 LRs, one or more models resulted in an LR < 1, while one or more other models showed support for the prosecution’s hypothesis. For example, in a 0.03ng 1-person sample (Figs 3 and 4), the contributor was not included in models A, B and C which consider forward stutter and included under Model D, which does not incorporate forward stutter. Further investigation revealed that this happened because at locus D16S539, the allele peak had a height of 6 RFU and the peak in the forward stutter position also had a height of 6 RFU, causing a 100% forward stutter ratio, which had a low probability. Though in reality, one or both of these peaks may contain significant levels of noise, or a combination of noise and signal, or noise and stutter, it is impossible to discern the precise contribution of signal, noise and stutter to the total fluorescence at any position. In the four models used in this study we have separate probabilistic models for the total height of peaks in allele, reverse stutter, forward stutter and noise positions. The opposite effect occurred for the minor contributor–Contributor 1 (starting template mass: 0.05ng) in a 1ng, 1:19 2-person sample (Fig 5), where the individual had an LR < 1 under Model D but had an LR > 1 under the other three models because inclusion of forward stutter gave a better explanation for the heights of the peaks at reverse and forward stutter position at the CSF1PO and vWA loci, since reverse stutter alone was not sufficient to explain the peak heights.

**Fig 6:**
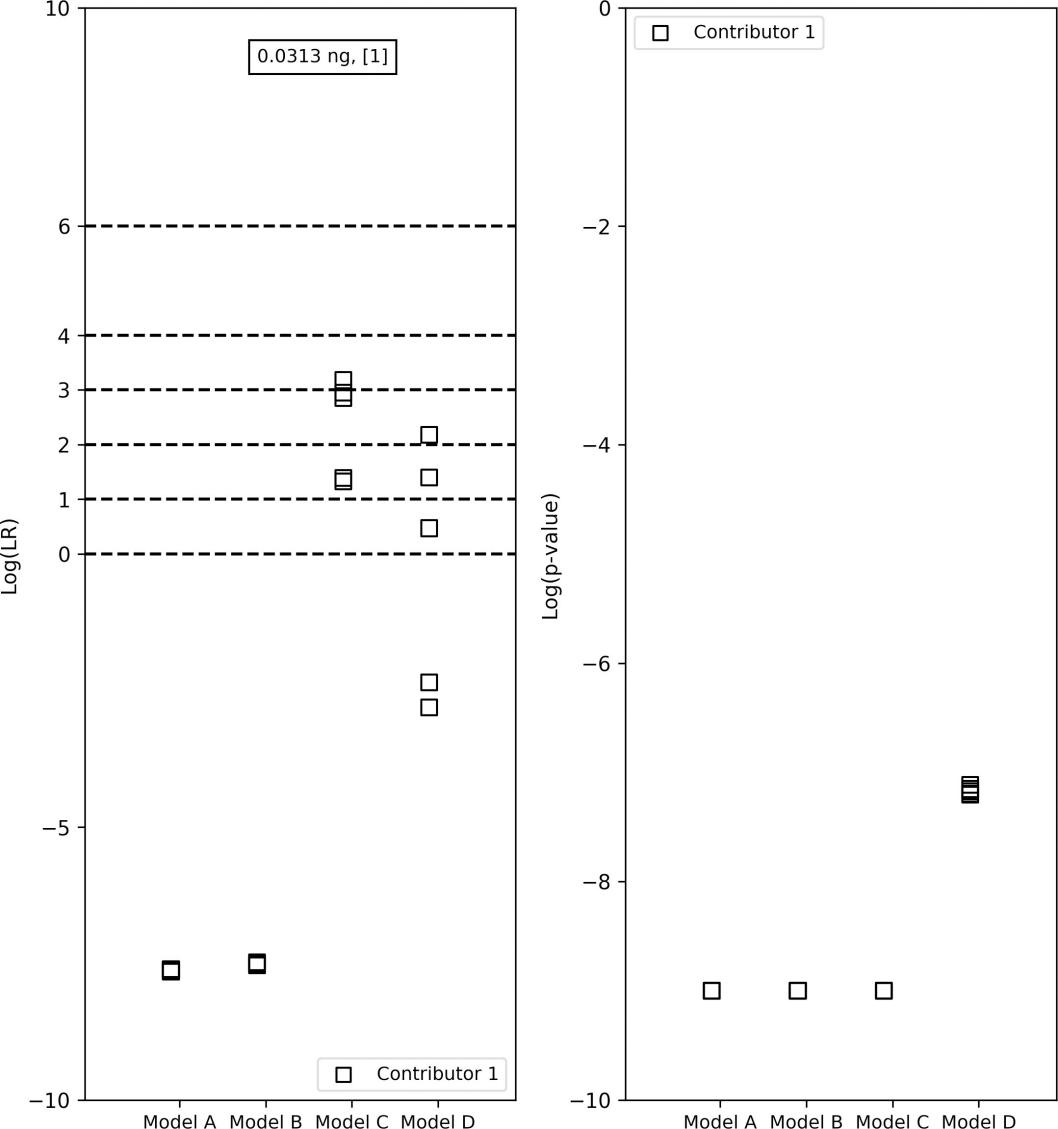
LR verbal expression levels and the LRs and *p*-values from the four models for the true contributor in a 0.03ng 1-person sample. Models A and B (normal noise distribution assumption) had LRs lower than 10^−7^ while Model C (lognormal noise distribution assumption) suggested ‘Strong’, ‘Moderately strong’ and ‘Moderate’ interpretations. LRs for Model D, fell both above and below 1 (log_10_(LR)s ranging from -2.81 to 2.18).

The lognormal assumption for the noise peak heights distribution is also an important one and had an effect on the interpretation. In a 0.03ng 1-person sample (Fig 6), Models A and B (which assume that the noise peak heights have a normal distribution) had LRs < 1 (lower than 10^−7^) while Model C (which has a lognormal noise distribution assumption) had LRs > 1 and suggested ‘Strong’, ‘Moderately strong’ and ‘Moderate’ interpretations. LRs for Model D, which also assumes a lognormal noise distribution but ignores the occurrence of forward stutter peaks, fell both above and below 1 (log_10_(LR)s ranging from -2.81 to 2.18). This occurred because even though the LR numerator was similar for all the versions, the LR denominator was much larger for Models A and B compared to Models C and D. Recall that the denominator of the LR is computed as:

$$\mathrm{Pr}\left(E|R=s,N=n\right)\mathrm{Pr}\left(R=s\right)+\frac{Pr(R\in R_{1}/\{s\})}{M}\sum_{i=1}^{M}\mathrm{Pr}\left(E|R=r^{i},N=n\right),$$

where *E* is the evidence (consisting of the peak heights observed in the signal), *M* is the number of random genotypes sampled (in this case 10^9^) and where ***R***_**1**_ is the set from which genotypes are sampled and consists of all genotypes *r* such that {Pr(*E*_*l*_|*R*_*l*_ = *r*_*l*_) ≄ 0 for all loci *l*}. In models A and B, there were only a few genotypes belonging to the set ***R***_**1**_ but the ones that did had a significantly large probability, resulting in a small value for Pr(*R* ∈ ***R***_**1**_) and a large summation term. The opposite occurred in versions C and D—a large value for Pr(*R* ∈ ***R***_**1**_) and a small value for the summation term—leading to a smaller overall value.

**Fig 7:**
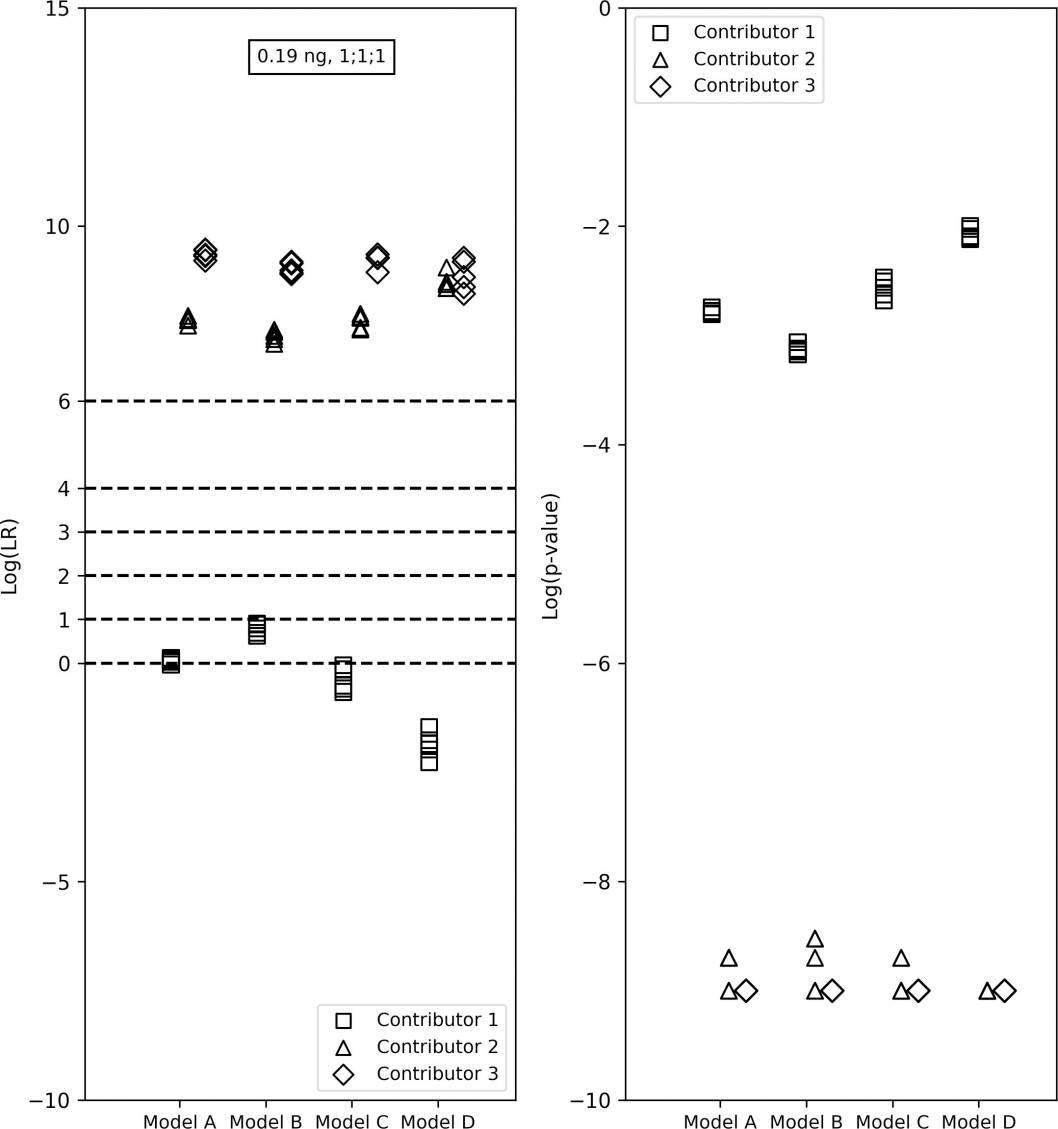
LR verbal expression levels and the LRs and *p*-values from the four models for the true contributors in a 1:1:1, 0.047ng 3-person sample. Models C and D (constant mixture ratio assumption) had LRs < 1. Model B (varying mixture ratio assumption) suggested ‘Weak’ interpretation. In Model A, the LRs fell both above and below 1 (log_10_(LR)s ranging from -0.03 to 0.11).

Finally, for Contributor 1 (starting template mass: 0.063ng) in a 3-person sample with 0.19ng of total template mass and a 1:1:1 mixture ratio (Fig 7), the LRs from the different versions were close to each other and also close to 1. Models C and D (both of which have a constant mixture ratio assumption) resulted in LRs < 1. Model B (which has a varying mixture ratio assumption) suggested that the evidence showed ‘Weak’ support for the prosecution’s hypothesis. In Model A, which also has a constant mixture ratio assumption but includes forward stutter peaks, the LRs fell both above and below 1 (log_10_(LR)s ranging from -0.03 to 0.11).

## Discussion

Given the extensive usage and reporting of DNA evidence to the courts, calculation and interpretation of the match statistic has substantive implications to criminal justice policy and practice. While the LR has gained precedence over the RMNE approach, the proliferation of continuous systems that compute the LR using different underlying model assumptions warrants an investigation into the final outcomes acquired from various models.

In addition, there is interest in evaluating and reporting the nature and source of variations between mixture interpretation protocols. Previous work on the subject has demonstrated significant differences in the mixture interpretation results between laboratories [40]. Specifically, the study demonstrated large differences in the LR reported by the laboratories that utilize them. For example, for Case 4 discussed in [40], in which the profile was generated from a two-person mixture with a minor contributor, laboratories that calculated a modified Random Match Probability (mRMP) or an LR reported statistics ranging from (1 in) 358,000 to 412 quintillion. Bille et al. [10] demonstrated that as the models evolve from binary to semi-continuous to continuous, so does the power of discrimination. It has also been shown that factors such as PCR and the content of allele frequency databases have an impact on the variation in the LR computed using a continuous method [28, 29]. It would be of benefit if the LR computed using a continuous method does not change significantly depending on the underlying model; however, we have observed in this study that variants of a continuous system, CEESIt, impact the LR and the subsequent verbal classification of some low template contributors.

In all four continuous models considered here and computed within the CEESIt framework, the verbal expression associated with the LR increased with an increase in the number of contributors and with a decrease in the contributor’s template mass. This corroborates the findings detailed in [28] and demonstrates that among the different sources of variation in the LR, uncertainty in genotype weight distributions can dominate the LR variation if the weights for the relevant genotypes are small. Significantly, there were differences between the models with respect to the upper limit of a contributor’s DNA mass below which intra-model variation was observed. All four models resulted in different verbal reports in the 2-person samples in instances where the contributor had less than 0.05ng of DNA except Model D, for which intra-model variability was also observed for one sample where contributor’s template DNA mass was in the range 0.05ng—0.15ng. However, in the 3-person samples, all four models exhibited intra-model differences in verbal classes when the contributor’s template DNA mass was less than 0.15ng except Model B, for which intra-model variability was also observed for one sample where contributor’s template DNA mass was more than 0.15ng (see Fig 1).

In addition to different verbal classifications within a model, inter-model verbal differences was also observed in this study. The four model variants examined in this study differed by one or two assumptions, and 32 out of the 195 LRs interrogated resulted in distinct verbal classifications across the models compared. Of these 32, 11 resulted in a change from LR < 1 to LR > 1 for contributors with low template masses. Notably, the models also differed in the type of mixtures in which they supported the defense’s hypothesis when tested against a true contributor to the sample. Model D resulted in an LR > 1 for contributors to mixtures, while the other three models resulted in an LR < 1 for single source samples and mixtures.

Verbal expressions of the LR are prone to misunderstanding and cannot be coherently combined with other evidence [41, 42]. Moreover, changing the LR verbal scale can cause a change in the way the numerical LR is communicated to the trier-of-fact. While we do not advocate their usage, they are employed in practice and thus the present paper employs verbal scales to demonstrate how LR variation between models potentially impacts the testimony of different experts.

The findings of this study have implications for the usage of, and communications associated with, probabilistic genotyping systems. As forensic laboratories implement probabilistic genotyping systems, characterizing the sensitivity of the LR to model assumptions of a continuous mixture interpretation method is necessary. Model differences and modifications are expected as these systems mature. The results of this paper suggest that any updated version of existing mixture interpretation software be tested on a large number of known samples to establish the range in which the system is deemed to be reliable and to verify that its results conform to expectations. Moreover, if the software is intended to be applied to low template samples, performing validation studies on such samples would inform the analyst as to the LRs typically obtained for such samples.

The mixture interpretation process can be thought of as a binary hypothesis test in which the hypotheses are as follows:

1. Null hypothesis (corresponding to the defense hypothesis *H*_*d*_) = A random, unknown person is the contributor to the sample.
2. Alternative hypothesis (corresponding to the prosecution hypothesis *H*_*p*_) = The person of interest is the contributor to the sample.

The LR is a statistic that expresses how many times more likely the data are under one hypothesis than the other. However, a large LR does not necessarily mean that the person of interest is a contributor, nor does a small LR preclude the person of interest from being a contributor, since the LR is sensitive to the quality of the data as well as to assumptions on the dropout probability, number of contributors, etc. [25–27]. The data presented herein demonstrate that for certain samples, the LR varied to a degree that affected a verbal classification based on the model used. The *p-*value of the LR is a summary statistic of the LR distribution conditioned on the defense hypothesis: it is the probability that a randomly chosen individual has an LR at least as large as the person of interest’s LR. One informative aspect of the *p-*value is that if the classification of individuals as contributors or non-contributors based on the *p-*value is that it allows control of the Type I error rate, or False Positive Rate (FPR). The FPR is the probability of incorrectly rejecting the null hypothesis when it is true and misclassifying the person of interest as a contributor.

Algorithms have been laid out for the computation of the LR distribution and the *p-*value [43, 44], but the *p-*value has faced its share of criticism as a statistic to replace the LR [38, 45]. In addition to enabling control of the FPR, a benefit of the *p-*value is that it can be used as an indicative tool while performing validation studies on a mixture interpretation system that computes the LR. The *p-*value can be used in conjunction with the LR to alert the developer or scientist to an LR that might be misleading due to the effect of the model assumptions. For example, for the minor contributor in the 2-person, 1ng, 1:19 sample in Fig 5, model D resulted in an LR < 1 while the other models resulted in LRs > 1. Correspondingly, the *p-*value from model D (10^−5^) was larger than the *p-*values from the other models (10^−8^ to 10^−9^), corroborating the LR interpretation. Conversely, in the 1-person, 0.03ng sample in Fig 6, models A and B resulted in small LRs < 1 that favored the defense’s hypothesis, while model C favored the prosecution’s hypothesis with LRs > 1 and model D had LRs both above and below 1. However, the *p*-values from models A and B were very small (10^−9^ is the upper bound), because while the genotype of the true contributor did not fit the signal well, based on the assumptions of models A and B, it was still a better explanation of the signal compared to the other random genotypes sampled. Though we do not necessarily recommend presenting the *p-*value in addition to, or instead of, the LR like [38], this study demonstrates that it can be beneficial to evaluate this statistic when performing validation studies on a continuous mixture interpretation software.

Lastly, we present the impact of model changes to verbal class, which have been presented alongside the numeric LR value computed by probabilistic system, suggesting that implementation of an updated version or distinct forensically relevant probabilistic system would require evaluation to ensure that its performance is compatible with existing interpretation protocols and verbal classification schemes, if used. In lieu of a verbal scale the use of hypothetical examples or calculation have been suggested. Given that four model variants of a single framework resulted in different verbal classes for some low-template contributors, additional studies that continue to examine possible sources of variability in LR outcomes and the methods by which forensic scientists communicate these finding are relevant to the forensic sciences and criminal justice practice.

## Conclusions

In addition to reducing the subjectivity associated with threshold-based schemes, forensically relevant continuous DNA genotyping systems are potentially powerful since they examine all or most of the information in the signal. In this paper, we studied the impact on the LR of changing a continuous model by using four different, but closely related variants of a continuous method. The four models were tested on 101 1-, 2- and 3-person experimental samples and the LR was computed to the true contributors to the samples. In all four models, intra-model variability in the LRs increased with an increase in the number of contributors and with a decrease in the contributor’s template mass. Within a forensic pipeline that includes verbal classifiers as a means to present LRs to the court, 32 of the 195 LRs resulted in LRs always differed by more than one verbal bin. Moreover, in 11 of these profiles there was a change from LR > 1 to LR < 1 for low-template contributors. The findings of this study underscore the importance of characterizing the variability in LR outcomes across genotyping systems using large-scale data to obtain and full and broad understanding of how LRs can change based on model, laboratory, threshold and verbal reporting decisions. Further, they show that new versions of a probabilistic genotyping models be validated using common validation procedures [46] and to confirm that modifications to the complementary verbal classification schemes are not required, if used. These data also bring to the fore potential limitations associated with attempts to bin LRs into categories.

## Supporting information

S1 Table Calibration Set–single source samples with known genotypes. (DOCX)

S2 Table Testing Set– 1, 2- and 3-person samples. (DOCX)

S3 Table The mixture ratios used to create the samples in the testing set and the mixture ratios used in the algorithm of the four continuous models. (DOCX)

S4 Table The variables used in the study and the distribution used to model them as a function of DNA mass. (DOCX)

S1 Appendix Appendix. (DOCX)

S1 Fig Mean of log_10_(LR) for the true contributors to the samples in the testing set from five runs of the four models. In each plot, the slope *α* and the intercept *β* of the best fit linear regression line are shown along with the *x* = *y* line. If the LRs do not differ based on the model, the points in the graph would lie along the *x* = *y* line and the values for the slope and the intercept would be 1 and 0, respectively. It can be seen from the figure that the slope and intercept of the best fit line for the comparisons of Model A vs Model B (both assume a normal distribution for noise peak heights) and Model C vs Model D (both assume a lognormal distribution for noise peak heights) are closest to the slope and intercept of the *x* = *y* line.
(TIF)

S2 Fig Average against difference of mean of log_10_(LR) for the true contributors to the samples in the testing set from five runs of the four models. In each plot, the *y* = 0 line is shown. If the LRs do not differ based on the model, the points in the graph would lie along the *y* = 0 line. While in most cases, the difference between the mean log_10_(LR) is small between a pair of models, there are cases where it is large (more than a few orders of magnitude). We also see that, for any given pair of models, there appears to be no dependence of the difference between the mean log_10_(LR) and its average. There is one large outlier point in each plot that is not shown whose coordinate is reported separately.
(TIF)

S3 Fig Scatter plot of log_10_(LR) against log_10_(*p*-value) for all samples in the testing set from five runs of the four models. We observe that the *p*-values decreased with an increase in the LR (Spearman’s rho = -0.75). For *p*-values greater than 10^−9^, the *p*-value is upper bounded by 1/LR as expected. For *p*-values of 10^−9^ or lower, the reported value represents only an upper bound to the true *p-*value.
(TIF)
