# Supplementary material for: Four model variants within a continuous forensic DNA mixture interpretation framework: Effects on evidential inference and reporting
Source: PLoS One. 2018 Nov 20;13(11):e0207599. doi: 10.1371/journal.pone.0207599 (PMC6245789; doi:10.1371/journal.pone.0207599)
Supplement: S1 Table — (DOCX) [file pone.0207599.s001.docx]

**S1 Table. Calibration Set – single source samples with known genotypes.**

| DNA amount (ng) | Number of samples |
| --- | --- |
| 0.008 | 48 |
| 0.016 | 45 |
| 0.031 | 46 |
| 0.047 | 27 |
| 0.063 | 42 |
| 0.125 | 48 |
| 0.25 | 48 |
| 0.5 | 9 |
| Total | 313 |
