# Supplementary material for: Four model variants within a continuous forensic DNA mixture interpretation framework: Effects on evidential inference and reporting
Source: PLoS One. 2018 Nov 20;13(11):e0207599. doi: 10.1371/journal.pone.0207599 (PMC6245789; doi:10.1371/journal.pone.0207599)
Supplement: S2 Table — (DOCX) [file pone.0207599.s002.docx]

**S2 Table. Testing Set – 1, 2- and 3-person samples.**

| 1-person samples | | 2-person samples | | 3-person samples | |
| --- | --- | --- | --- | --- | --- |
| DNA amount (ng) | Number of samples | DNA amount (ng) | Number of samples | DNA amount (ng) | Number of samples |
| 0.016 | 5 | 0.031 | 3 | 0.04 | 1 |
| 0.031 | 4 | 0.047 | 4 | 0.05 | 1 |
| 0.047 | 5 | 0.063 | 6 | 0.1 | 9 |
| 0.063 | 5 | 0.125 | 9 | 0.2 | 11 |
| 0.125 | 5 | 0.25 | 12 | 0.3 | 3 |
| 0.25 | 5 | 0.5 | 5 | 0.4 | 2 |
| 1 | 1 | 1 | 2 | 0.5 | 1 |
|  |  |  |  | 0.6 | 2 |
| Total | 30 | Total | 41 | Total | 30 |
