# Supplementary material for: Four model variants within a continuous forensic DNA mixture interpretation framework: Effects on evidential inference and reporting
Source: PLoS One. 2018 Nov 20;13(11):e0207599. doi: 10.1371/journal.pone.0207599 (PMC6245789; doi:10.1371/journal.pone.0207599)
Supplement: S3 Table — (DOCX) [file pone.0207599.s003.docx]

**S3 Table. The mixture ratios used to create the samples in the testing set and the mixture ratios used in the algorithm of the four continuous models.**

| Number of contributors | Mixture ratios used to create the samples in the testing set | Mixture ratios used in the algorithm of the four models |
| --- | --- | --- |
| 2 | 1:1, 1:2, 1:4, 1:9, 1:19 | 1:9, 1:4, 3:7, 2:3, 1:1, 3:2, 7:3, 4:1, 9:1 |
| 3 | 1:1:1, 1:1:2, 1:1:4, 1:1:9, 1:2:2, 1:4:4, 1:9:9 | 3:2:1, 2:1:3, 1:2:3, 6:2:1, 1:6:2, 2:1:6, 9:1:1, 1:9:1, 1:1:9, 5:1:3, 3:5:1, 1:3:5 |
