# Supplementary material for: Four model variants within a continuous forensic DNA mixture interpretation framework: Effects on evidential inference and reporting
Source: PLoS One. 2018 Nov 20;13(11):e0207599. doi: 10.1371/journal.pone.0207599 (PMC6245789; doi:10.1371/journal.pone.0207599)
Supplement: S4 Table — (DOCX) [file pone.0207599.s004.docx]

**S4 Table. The variables used in the study and the distribution used to model them as a function of DNA mass.**

| Variable | Model description | Distribution of the variable |
| --- | --- | --- |
| Dropout rate of alleles ($\alpha)$ | Exponentially decreasing curve | $\alpha=ae^{bx}$, where $x$ is the DNA mass from the contributor with the allele |
| Rate of non-occurrence of stutter ($\beta)$ | Exponentially decreasing curve | $\beta=ae^{bx}$, where $x$ is the DNA mass in the parent peak that gives rise to stutter |
| Rate of non-detection of noise ($\gamma$) | Constant | Estimated from calibration data |
| Mean of true peak heights ($\mu_{t}$) | Line with a positive slope | $\mu_{t}=ax+b$, where $x$ is the DNA mass in the true peak |
| Standard deviation of true peak heights ($\sigma_{t}$) | Line with a positive slope | $\sigma_{t}=ax+b$, where $x$ is the DNA mass in the true peak |
| Mean of noise peak heights ($\mu_{n}$) | Line with a positive slope | $\mu_{n}=ax+b$, where $x$ is the DNA mass that the sample was amplified with |
| Standard deviation of noise peak heights ($\sigma_{n}$) | Line with a positive slope | $\sigma_{n}=ax+b$, where $x$ is the DNA mass that the sample was amplified with |
| Mean of stutter ratios ($\mu_{s}$) | Exponentially decreasing curve | $\mu_{s}=ae^{bx}+c$, where $x$ is the DNA mass in the parent peak that gives rise to stutter |
| Standard deviation of stutter ratios ($\sigma_{s}$) | Exponentially decreasing curve | $\sigma_{s}=ae^{bx}+c$, where $x$ is the DNA mass in the parent peak that gives rise to stutter |
