# Supplementary material for: Four model variants within a continuous forensic DNA mixture interpretation framework: Effects on evidential inference and reporting
Source: PLoS One. 2018 Nov 20;13(11):e0207599. doi: 10.1371/journal.pone.0207599 (PMC6245789; doi:10.1371/journal.pone.0207599)
Supplement: S1 Appendix — (DOCX) [file pone.0207599.s005.docx]

**APPENDIX**

The problem at hand is to compute $Pr(E_{l}|\boldsymbol{G=g,}\boldsymbol{\Theta}=\boldsymbol{\theta}, N=n)$, which is the probability of observing the evidence at a locus $l$, given the mixture proportions, the genotypes of the contributors and the number of contributors. Each of these terms is described in detail below:

$N$ is a positive integer that represents the number of contributors.

$\boldsymbol{G}$ is a vector with components $G_{i}$, the genotype of each contributor $i\in\left\{ 1,\ldots,n \right\}$:

$$\boldsymbol{G=}(G_{1},\ldots,G_{n}).$$

$\boldsymbol{\Theta}$ is an $n$-dimensional vector with components $\Theta_{i}$, the mixture proportion of each contributor $i\in\left\{ 1,\ldots,n \right\}$:

$\boldsymbol{\Theta=(}\Theta_{1},\Theta_{2},\ldots\Theta_{n}):\sum_{i=1}^{n} \Theta_{i}=1\wedge\Theta_{i}\in\mathbb{R}_{>0}\forall i=1,\ldots n$,

where $\mathbb{R}_{>0}$ refers to the set of real numbers greater than 0.

We use the vector $\boldsymbol{a}$ to represent the alleles in the genotypes of the contributors:

$$\boldsymbol{a}=(a_{1},\ldots,a_{2n}),$$

where for $i=1,\ldots,2n$, allele $a_{i}$ belongs to $G_{\left\lceil i/2 \right\rceil}$, the genotype of contributor $\left\lceil i/2 \right\rceil$.

At all the autosomal STR loci, allele $a_{i}\mathcal{\in A \forall}i=1,\ldots,2n,$ where $\mathcal{A}$ is a finite set of real numbers that represents all possible alleles (i.e. numbers of repeats), e.g., 9, 9.3, 10, etc., for the STRs at locus $l.$ At the sex-determining locus AMEL, allele $a_{i}\in\left\{ X,Y \right\}.$

To describe the signal $E_{l}$, we first define $\boldsymbol{x}$ to be the set that contains all repeat values of STRs at locus $l$ that correspond to allele, reverse stutter and forward stutter positions:

$$\boldsymbol{x}\mathcal{=A\cup}\left\{ a-1:a\in\mathcal{A} \right\}\cup\left\{ a+1:a\in\mathcal{A} \right\}.$$

At the AMEL locus, the set $\boldsymbol{x}$ comprises of two alleles: X and Y.

Now, we can represent the evidence $E_{l}$ as a $|\boldsymbol{x}|$-dimensional vector $\boldsymbol{H\in}\mathbb{Z}_{\boldsymbol{\geq0}}^{|\boldsymbol{x}|}$ that consists of the peak heights observed in the signal, which are non-negative integers:

$$E_{l}\boldsymbol{=H=}\left( h_{x}:x\in\boldsymbol{x}\bigwedge h_{x}\in\mathbb{Z}_{\geq0} \right),$$

where $h_{x}$ is the height of allele $x$. We can now condition the probability of observing the evidence upon $\boldsymbol{A}$ instead of $\boldsymbol{G}$:

$$\Pr\left( E_{l} | \boldsymbol{G=g,}\boldsymbol{\Theta}=\boldsymbol{\theta}, N=n \right)=\sum_{\boldsymbol{d}\in\boldsymbol{D}} \Pr\left( E_{l} | \mathbf{D}\boldsymbol{=d,A=a,}\boldsymbol{\Theta}=\boldsymbol{\theta}, N=n \right)\Pr\left( \mathbf{D}\boldsymbol{=d} \right),$$

where $\boldsymbol{D}$ is a set that comprises of all possible dropout combinations for the alleles in the signal: $\boldsymbol{D=}\left\{ \left( D_{1},\ldots,D_{2n} \right) \right\},$ where for $i=1,\ldots,2n, D_{i}=\left\{ \begin{aligned} 0, &\text{if allele} a_{i}\text{ dropped out,} \\ 1, & \text{otherwise.} \end{aligned} \right.$

Next, we define two sets $\hat{\boldsymbol{a}}$ and $\boldsymbol{m}$ that the signal $E_{l}$ depends upon. Let $\hat{\boldsymbol{a}}$ be the set of all alleles in the genotypes of the contributors that have not dropped out:

$\hat{\boldsymbol{a}}=\bigcup_{i=1}^{2n} a_{i}:D_{i}=1$.

Let the total template DNA mass be *M*. Each allele $a\in\hat{\boldsymbol{a}}$ in the genotype of the contributors has some mass $m_{a}$ associated with it, that is the sum of the template masses of all the contributors who have that allele without drop out:

$$m_{a}=\sum_{1\leq i\leq2n:a_{i}=a\wedge D_{i}=1} {M*\Theta}_{\left\lceil i/2 \right\rceil}.$$

We define $\boldsymbol{m}$ to be the vector containing all the masses of the alleles: $\boldsymbol{m=(}m_{a}\boldsymbol{:}a\boldsymbol{\in}\hat{\boldsymbol{a}}).$

The term $\Pr\left( \mathbf{D}\boldsymbol{=d} \right)$ is computed using the dropout probability model as described in S4 Table using the template masses of the contributors. It is now sufficient to condition the evidence $E_{l}$ upon the set of alleles $\hat{\boldsymbol{a}}$ and their masses $\boldsymbol{m}$, since the information captured in them is enough to compute the probability of observing the peak heights:

|  | $\Pr\left( E_{l} \vert\mathbf{D}\boldsymbol{=d,A=a,}\boldsymbol{\Theta}=\boldsymbol{\theta}, N=n \right) =Pr \left( \boldsymbol{H=h} \vert\hat{\boldsymbol{A}}=\hat{\boldsymbol{a}}\boldsymbol{,M}\mathbf{=}\boldsymbol{m} \right)$ | (1) |
| --- | --- | --- |

We have following two conditions based on our dropout model, where we assume that an allele that has not dropped out must be observed (i.e. have a height > 0) and if an allele is observed then it must not have dropped out:

$\Pr\left( \boldsymbol{H=h} | \hat{\boldsymbol{A}}=\hat{\boldsymbol{a}}\boldsymbol{,M}\mathbf{=}\boldsymbol{m} \right)=0$if $h_{a}=0$for any $a\in\hat{\boldsymbol{a}}$;

$\Pr\left( \boldsymbol{H=h} | \hat{\boldsymbol{A}}=\hat{\boldsymbol{a}}\boldsymbol{,M}\mathbf{=}\boldsymbol{m} \right)=0$ if $h_{a}>0 \wedge$ $a\notin\hat{\boldsymbol{a}}$ for any $a\in\boldsymbol{a}$.

Let $\hat{\boldsymbol{r}}$ and $\hat{\boldsymbol{f}}$ be the sets containing the alleles in the reverse and forward positions of observed alleles in $\hat{\boldsymbol{a}}$, respectively:

$$\hat{\boldsymbol{r}}=\left\{ a-1:a\in\hat{\boldsymbol{a}} \bigwedge h_{a}>0 \right\};$$

$$\hat{\boldsymbol{f}}=\left\{ a+1:a\in\hat{\boldsymbol{a}} \bigwedge h_{a}>0 \right\}.$$

Model D does not consider forward stutter and hence $\hat{\boldsymbol{f}}$ is not used in model D. We make the simplifying assumption that when forward stutter is present along with one or more alleles, the effect of forward stutter is masked by the allele(s) and its contribution to the peak height is negligible. Hence, a combination of allele and forward stutter is effectively treated as being exclusively allelic in origin. One way to refine our model would be to remove this simplifying assumption and incorporate forward stutter into the calculation even when it is combined with one or more alleles.

We define the following sets as part of our model:

$\boldsymbol{a=}\hat{\boldsymbol{a}} \backslash\hat{\boldsymbol{r}}$ is the set containing alleles that are in the genotype of the contributors and have no stutter effects (the contribution of forward stutter to an allelic peak is ignored);

$\boldsymbol{r=}\hat{\boldsymbol{r}}\setminus(\hat{\boldsymbol{a}}\cup\hat{\boldsymbol{f}})$ is the set containing alleles with only reverse stutter contributions;

$\boldsymbol{f=}\hat{\boldsymbol{f}}\setminus(\hat{\boldsymbol{a}}\cup\hat{\boldsymbol{r}})$ is the set containing alleles with only forward stutter contributions;

$\boldsymbol{t=}\hat{\boldsymbol{a}}\cap\hat{\boldsymbol{r}}$ is the set containing alleles with allelic and reverse stutter contributions (again, the contribution of forward stutter in the presence of alleles is ignored);

$\boldsymbol{u=(}\hat{\boldsymbol{r}}\cap\hat{\boldsymbol{f}})\setminus\hat{\boldsymbol{a}}$ is the set containing alleles with reverse and forward stutter contributions;

$\boldsymbol{b}=\boldsymbol{x}\setminus(\hat{\boldsymbol{a}}\cup\hat{\boldsymbol{r}}\cup\hat{\boldsymbol{f}})$ is the set containing all the baseline noise alleles.

Sets $\boldsymbol{f}$ and $\boldsymbol{u}$ are not utilized in model D, which does not consider forward stutter. For this version, the set $\boldsymbol{r}$ is simply $\hat{\boldsymbol{r}}$ and $\boldsymbol{b}=\boldsymbol{x}\setminus(\hat{\boldsymbol{a}}\cup\hat{\boldsymbol{r}})$.

All the alleles that give rise to the signal can be classified under one of the six sets defined above. The set $\boldsymbol{x}$ can be expressed as the disjoint union of these six sets:

$$\boldsymbol{x=a+r+f+t+u+b.}$$

Since stutter is not possible at the AMEL locus, the signal can be described using two sets:

$$\boldsymbol{x=a+b.}$$

Since the set $\boldsymbol{x}$ comprises of all the alleles giving rise to the signal, we have:

| $\Pr\left( \boldsymbol{H=h} \vert\hat{\boldsymbol{A}}=\hat{\boldsymbol{a}}\boldsymbol{,M}\mathbf{=}\boldsymbol{m} \right)$ $=\Pr\left( \bigcup_{x\in\boldsymbol{x}} H_{x}=h_{x}\vert\hat{\boldsymbol{A}}=\hat{\boldsymbol{a}}\boldsymbol{,M}\mathbf{=}\boldsymbol{m} \right)$ $=\Pr\left( \bigcup_{a\in\boldsymbol{a}} H_{a}=h_{a}\vert\hat{\boldsymbol{A}}=\hat{\boldsymbol{a}}\boldsymbol{,M}\mathbf{=}\boldsymbol{m} \right)\times Pr(\bigcup_{x\in(\boldsymbol{x\backslash a)}} H_{x}=h_{x}\left\vert\bigcup_{a\in\boldsymbol{a}} H_{a}=h_{a},\hat{\boldsymbol{A}}=\hat{\boldsymbol{a}}\boldsymbol{,M}\mathbf{=}\boldsymbol{m} \right)$ | (2) |
| --- | --- |

The term $Pr(\bigcup_{a\in\boldsymbol{a}} H_{a}=h_{a}\left| \hat{\boldsymbol{A}}=\hat{\boldsymbol{a}}\boldsymbol{,M}\mathbf{=}\boldsymbol{m} \right)$ is the probability of observing the heights of peaks that contain only allelic contributions and no stutter effects. The probability of the heights of these peaks depends only upon the mass of the peak and not upon the other alleles or their masses. Hence we have:

$$Pr(\bigcup_{a\in\boldsymbol{a}} H_{a}=h_{a}\left| \hat{\boldsymbol{A}}=\hat{\boldsymbol{a}}\boldsymbol{,M}\mathbf{=}\boldsymbol{m} \right)=\prod_{a\in\boldsymbol{a}} Pr(H_{a}=h_{a}\left| M_{a}=m_{a} \right)\boldsymbol{.}$$

The peak heights in the signal are expressed in RFUs as integers and hence take discrete values. However, we have modeled the peak heights using the normal distribution, which is a continuous distribution. Therefore, we approximate the probability of observing a peak at a particular height as the density of the normal distribution at that height. Let $p$ and $P$ be the PDF and the CDF of the normal distribution, respectively. If $\alpha>0$ is the height of an allelic peak with mass $m_{\alpha}$, then the probability of the peak having height $\alpha$ is approximated as:

$$P\left( \alpha+0.5;\mu_{m_{\alpha}},\sigma_{m_{\alpha}} \right)-P\left( \alpha-0.5;\mu_{m_{\alpha}},\sigma_{m_{\alpha}} \right)=\int_{\alpha-0.5}^{\alpha+0.5} p\left( \alpha;\mu_{m_{\alpha}},\sigma_{m_{\alpha}} \right) d\alpha$$

$$\cong p\left( \alpha;\mu_{m_{\alpha}},\sigma_{m_{\alpha}} \right),$$

where $\mu_{m_{\alpha}}$ and $\sigma_{m_{\alpha}}$ are the mean and standard deviation that correspond to a mass of $m_{\alpha}$. The dependence of the mean and standard deviation on the DNA mass of the allele is specified in S4 Table.

|  | $Pr(H_{a}=h_{a}\left\vert M_{a}=m_{\boldsymbol{a}} \right)\boldsymbol{=}p\left( h_{a};\mu_{m_{a}},\sigma_{m_{a}} \right)$ | (3) |
| --- | --- | --- |

The term $Pr(\bigcup_{x\in(\boldsymbol{x\backslash a)}} H_{x}=h_{x}\left| \bigcup_{a\in\boldsymbol{a}} H_{a}=h_{a},\hat{\boldsymbol{A}}= \hat{\boldsymbol{a}}\boldsymbol{,M}\mathbf{=}\boldsymbol{m} \right)\boldsymbol{,}$ the second term in equation (2), is the probability of observing the heights of all the peaks other than the ones in $\boldsymbol{a}$. This can be written as:

| $Pr(\bigcup_{x\in(\boldsymbol{x\backslash a)}} H_{x}=h_{x}\left\vert\bigcup_{a\in\boldsymbol{a}} H_{a}=h_{a},\hat{\boldsymbol{A}}= \hat{\boldsymbol{a}}\boldsymbol{,M}\mathbf{=}\boldsymbol{m} \right)$  = $\Pr\left( \bigcup_{r\in\boldsymbol{r}} H_{r}=h_{r} \vert\bigcup_{a\in\boldsymbol{a}} H_{a}=h_{a},\hat{\boldsymbol{A}}= \hat{\boldsymbol{a}}\boldsymbol{,M}\mathbf{=}\boldsymbol{m} \right)$  $\times\Pr\left( \bigcup_{f\in\boldsymbol{f}} H_{f}=h_{f} \vert\bigcup_{r\in\boldsymbol{r}} H_{r}=h_{r},\bigcup_{a\in\boldsymbol{a}} H_{a}=h_{a},\hat{\boldsymbol{A}}= \hat{\boldsymbol{a}}\boldsymbol{,M}\mathbf{=}\boldsymbol{m} \right)$ $\times\Pr\left( \bigcup_{x\in(\boldsymbol{x\setminus}\left( \boldsymbol{a\cup r\cup f} \right)\boldsymbol{)}} H_{x}=h_{x} \vert\begin{aligned} \bigcup_{f\in\boldsymbol{f}} H_{f}=h_{f},\bigcup_{r\in\boldsymbol{r}} H_{r}=h_{r}, \\ \bigcup_{a\in\boldsymbol{a}} H_{a}=h_{a},\hat{\boldsymbol{A}}= \hat{\boldsymbol{a}}\boldsymbol{,M=m}) \end{aligned} \right)$ | (4) |
| --- | --- |

The term $\Pr\left( \bigcup_{r\in\boldsymbol{r}} H_{r}=h_{r} | \bigcup_{a\in\boldsymbol{a}} H_{a}=h_{a},\hat{\boldsymbol{A}}= \hat{\boldsymbol{a}}\boldsymbol{,M}\mathbf{=}\boldsymbol{m} \right)$ is the probability of observing the reverse stutter peaks. Conditioned upon the allele heights, the stutter peak heights are independent of each other:

$\Pr\left( \bigcup_{r\in\boldsymbol{r}} H_{r}=h_{r} | \bigcup_{a\in\boldsymbol{a}} H_{a}=h_{a},\hat{\boldsymbol{A}}= \hat{\boldsymbol{a}}\boldsymbol{,M}\mathbf{=}\boldsymbol{m} \right)$

$$=\prod_{r\in\boldsymbol{r}} Pr(H_{r}=h_{r}\left| \bigcup_{a\in\boldsymbol{a}} H_{a}=h_{a},\hat{\boldsymbol{A}}=\hat{\boldsymbol{a}}\boldsymbol{,M}\mathbf{=}\boldsymbol{m} \right)\boldsymbol{.}$$

We have modeled stutter peak heights using the stutter ratio. Hence the height of a stutter peak depends upon the height of the parent peak. For a reverse stutter allele $r$, $r+1$ is the parent allele causing reverse stutter. Let $q_{stut}$ (which depends upon the mass of the parent allele and is described in S4 Table) be the rate of non-detection of stutter. To calculate the probability of a stutter peak height, we make the same approximation that we did for the allelic peaks, with the modification that the stutter peak height $H_{r}$ is coming from a distribution scaled according to the height of the parent peak:

$$H_{r}\mathcal{\sim N}\left( \mu h_{r+1},\sigma h_{r+1} \right).$$

We calculate the probability of observing the reverse stutter peak heights as follows:

| $Pr(H_{r}=h_{r}\vert\bigcup_{a\in\boldsymbol{a}} H_{a}=h_{a},\hat{\boldsymbol{A}}=\hat{\boldsymbol{a}}\boldsymbol{,M}\mathbf{=}\boldsymbol{m)}$  $=Pr(H_{r}=h_{r}\left\vert{H_{r+1}=h}_{r+1}\boldsymbol{,}{M_{r+1}=m}_{r+1} \right)$  $= \left\{ \begin{aligned} p\left( h_{r};h_{r+1}\mu_{m_{r+1}},{h_{r+1}\sigma}_{m_{r+1}} \right) \left( 1-q_{stut}\left( m_{r+1} \right) \right), &\text{if }h_{r}>0\text{,} \\ q_{stut}\left( m_{r+1} \right), &\text{otherwise.} \end{aligned} \right.$ | (5) |
| --- | --- |

The term $\Pr\left( \bigcup_{f\in\boldsymbol{f}} H_{f}=h_{f} | \bigcup_{r\in\boldsymbol{r}} H_{r}=h_{r},\bigcup_{a\in\boldsymbol{a}} H_{a}=h_{a},\hat{\boldsymbol{A}}= \hat{\boldsymbol{a}}\boldsymbol{,M}\mathbf{=}\boldsymbol{m} \right)$ in (4) corresponds to the probability of observing the forward stutter peak heights. We notice that the forward stutter heights do not depend upon the reverse stutter heights and are independent of each other conditioned on the heights of the alleles. Hence we get:

$$\Pr\left( \bigcup_{f\in\boldsymbol{f}} H_{f}=h_{f} | \bigcup_{r\in\boldsymbol{r}} H_{r}=h_{r},\bigcup_{a\in\boldsymbol{a}} H_{a}=h_{a},\hat{\boldsymbol{A}}= \hat{\boldsymbol{a}}\boldsymbol{,M}\mathbf{=}\boldsymbol{m} \right)$$

$$=\prod_{f\in\boldsymbol{f}} Pr\left( H_{f}=h_{f}|\bigcup_{a\in\boldsymbol{a}} H_{a}=h_{a},\hat{\boldsymbol{A}}= \hat{\boldsymbol{a}}\boldsymbol{,M}\mathbf{=}\boldsymbol{m} \right)\boldsymbol{.}$$

For a forward stutter allele $f$, $f-1$ is the parent allele causing forward stutter. We calculate the probability of observing the height of a forward stutter peak in a manner similar to that used for the reverse stutter heights:

| $Pr(H_{f}=h_{f}\vert\bigcup_{a\in\boldsymbol{a}} H_{a}=h_{a},\hat{\boldsymbol{A}}=\hat{\boldsymbol{a}}\boldsymbol{,M}\mathbf{=}\boldsymbol{m)}$  $=Pr(H_{f}=h_{f}\left\vert{H_{f-1}=h}_{f-1}\boldsymbol{,}{M_{f-1}=m}_{f-1} \right)$  $= \left\{ \begin{aligned} p\left( h_{f};h_{f-1}\mu_{m_{f-1}},{h_{f-1}\sigma}_{m_{f-1}} \right) \left( 1-q_{stut}\left( m_{f-1} \right) \right), &\text{if }h_{f}>0\text{,} \\ q_{stut}\left( m_{f-1} \right), &\text{otherwise.} \end{aligned} \right.$ | (6) |
| --- | --- |

$\Pr\left( \bigcup_{x\in(\boldsymbol{x\setminus}\left( \boldsymbol{a\cup r\cup f} \right)\boldsymbol{)}} H_{x}=h_{x} | \bigcup_{f\in\boldsymbol{f}} H_{f}=h_{f},\bigcup_{r\in\boldsymbol{r}} H_{r}=h_{r},\bigcup_{a\in\boldsymbol{a}} H_{a}=h_{a},\hat{\boldsymbol{A}}= \hat{\boldsymbol{a}}\boldsymbol{,M=m} \right)$ is the third term in equation (4) and represents the probability of observing the heights of all the peaks not in $\boldsymbol{a}$, $\boldsymbol{r}$ and $\boldsymbol{f}$. This probability is independent of the stutter peak heights and hence those terms can be eliminated:

$\Pr\left( \bigcup_{x\in(\boldsymbol{x\setminus}\left( \boldsymbol{a\cup r\cup f} \right)\boldsymbol{)}} H_{x}=h_{x} | \bigcup_{f\in\boldsymbol{f}} H_{f}=h_{f},\bigcup_{r\in\boldsymbol{r}} H_{r}=h_{r},\bigcup_{a\in\boldsymbol{a}} H_{a}=h_{a},\hat{\boldsymbol{A}}= \hat{\boldsymbol{a}}\boldsymbol{,M=m} \right)$

$$=\Pr\left( \bigcup_{x\in(\boldsymbol{x\setminus}\left( \boldsymbol{a\cup r\cup f} \right)\boldsymbol{)}} H_{x}=h_{x} | \bigcup_{a\in\boldsymbol{a}} H_{a}=h_{a},\hat{\boldsymbol{A}}= \hat{\boldsymbol{a}}\boldsymbol{,M=m} \right).$$

The set $\boldsymbol{x\setminus(a\cup r\cup f)}$ can be divided into three disjoint sets: $\boldsymbol{b}$, which contains the noise alleles;$\boldsymbol{u}$, which contains alleles that have reverse and forward stutter effects; and $\boldsymbol{t}$, which contains allele and reverse stutter effects.

| $\Pr\left( \bigcup_{x\in\left( \boldsymbol{x\setminus}\left( \boldsymbol{a\cup r\cup f} \right) \right)} H_{x}=h_{x} \vert\bigcup_{a\in\boldsymbol{a}} H_{a}=h_{a},\hat{\boldsymbol{A}}= \hat{\boldsymbol{a}}\boldsymbol{,M=m} \right)$ $=Pr \left( \bigcup_{t\in\boldsymbol{t}} H_{t}=h_{t} \vert\bigcup_{a\in\boldsymbol{a}} H_{a}=h_{a},\hat{\boldsymbol{A}}= \hat{\boldsymbol{a}}\boldsymbol{,M=m} \right)$  $\times\Pr\left( \bigcup_{u\in\boldsymbol{u}} H_{u}=h_{u} \vert\bigcup_{t\in\boldsymbol{t}} H_{t}=h_{t},\bigcup_{a\in\boldsymbol{a}} H_{a}=h_{a},\hat{\boldsymbol{A}}= \hat{\boldsymbol{a}}\boldsymbol{,M=m} \right)$  $\times\Pr\left( \bigcup_{b\in\boldsymbol{b}} H_{b}=h_{b} \vert\bigcup_{u\in\boldsymbol{u}} H_{u}=h_{u},\bigcup_{t\in\boldsymbol{t}} H_{t}=h_{t},\bigcup_{a\in\boldsymbol{a}} H_{a}=h_{a},\hat{\boldsymbol{A}}= \hat{\boldsymbol{a}}\boldsymbol{,M=m} \right)$ | (7) |
| --- | --- |

Notice that the heights of the noise alleles $\boldsymbol{b}$ depend only upon the template DNA mass $M$ and are independent of the heights of other peaks. Let $q_{\mathrm{noise}}$ be the probability of not detecting a noise allele. Hence we have:

$\Pr\left( \bigcup_{b\in\boldsymbol{b}} H_{b}=h_{b} | \bigcup_{u\in\boldsymbol{u}} H_{u}=h_{u},\bigcup_{t\in\boldsymbol{t}} H_{t}=h_{t},\bigcup_{a\in\boldsymbol{a}} H_{a}=h_{a},\hat{\boldsymbol{A}}= \hat{\boldsymbol{a}}\boldsymbol{,M=m} \right)$

$=\prod_{b\in\boldsymbol{b}} Pr(H_{b}=h_{b}\mathcal{|M=}M)$,

and

|  | $Pr(H_{b}=h_{b}\mathcal{\vert M=}M)= \left\{ \begin{aligned} p\left( h_{b};\mu_{M},\sigma_{M} \right) \left( 1-q_{\mathrm{noise}} \right), &\text{if }h_{b}>0\text{,} \\ q_{\mathrm{noise}}, &\text{otherwise.} \end{aligned} \right.$ | (8) |
| --- | --- | --- |

For models C and D, which assume that the noise peak heights have a lognormal distribution, the PDF of the lognormal distribution is used to compute the probability of the noise peak heights.

Next, we look at the second term in equation **(**7**)**. The set $\boldsymbol{u}$ is a combination of two events: reverse stutter and forward stutter. For an allele $u\in\boldsymbol{u}$, $u+1$ is the parent allele causing reverse stutter at $u$ and $u-1$ is the parent allele causing forward stutter at $u$. We assume that the two events reverse and forward stutter occur independent of each other. If a peak is observed, then both or any one of the two events might have occurred. On the other hand, if a peak is not observed, then there is a dropout of both events. Therefore we get:

$\Pr\left( \bigcup_{u\in\boldsymbol{u}} H_{u}=h_{u} | \bigcup_{t\in\boldsymbol{t}} H_{t}=h_{t},\bigcup_{a\in\boldsymbol{a}} H_{a}=h_{a},\hat{\boldsymbol{A}}= \hat{\boldsymbol{a}}\boldsymbol{,M=m} \right)$

$=\prod_{u\in\boldsymbol{u}} Pr(H_{u}=h_{u}\left| {H_{u+1}=h}_{u+1}\boldsymbol{,}M_{u+1}=m_{u+1}\boldsymbol{,}{H_{u-1}=h}_{u-1}\boldsymbol{,}M_{u-1}=m_{u-1} \right)$**,**

where, for $h_{u}>0$,

| ${Pr(H}_{u}=h_{u}\left\vert{H_{u+1}=h}_{u+1}\boldsymbol{,}M_{u+1}=m_{u+1}\boldsymbol{,}{H_{u-1}=h}_{u-1}\boldsymbol{,}M_{u-1}=m_{u-1} \right)$  $=p\left( h_{u};h_{u+1}\mu_{m_{u+1}}+h_{u-1}\mu_{m_{u-1}},{h_{u+1}\sigma}_{m_{u+1}}+{h_{u-1}\sigma}_{m_{u-1}} \right)$ $\times\left( 1-q_{stut}\left( m_{u+1} \right) \right)\left( 1-q_{stut}\left( m_{u-1} \right) \right)$ $+p\left( h_{u};h_{u+1}\mu_{m_{u+1}},{h_{u+1}\sigma}_{m_{u+1}} \right) \left( 1-q_{stut}\left( m_{u+1} \right) \right)q_{stut}\left( m_{u-1} \right)$ $+p\left( h_{u};h_{u-1}\mu_{m_{u-1}},{h_{u-1}\sigma}_{m_{u-1}} \right) \left( 1-q_{stut}\left( m_{u-1} \right) \right)q_{stut}\left( m_{u+1} \right)$ | (9a) |
| --- | --- |

and otherwise,

| ${Pr(H}_{u}=h_{u}\left\vert{H_{u+1}=h}_{u+1}\boldsymbol{,}M_{u+1}=m_{u+1}\boldsymbol{,}{H_{u-1}=h}_{u-1}\boldsymbol{,}M_{u-1}=m_{u-1} \right)$ $=q_{stut}\left( m_{u+1} \right)q_{stut}\left( m_{u-1} \right)$ | (9b) |
| --- | --- |

The other term to compute in (7) is $\Pr\left( \bigcup_{t\in\boldsymbol{t}} H_{t}=h_{t} | \bigcup_{a\in\boldsymbol{a}} H_{a}=h_{a},\hat{\boldsymbol{A}}= \hat{\boldsymbol{a}}\boldsymbol{,M=m} \right)$. $\boldsymbol{t}$ is the set containing alleles that have allele and reverse stutter effects. The probability of observing the height of an allele $t\in\boldsymbol{t}$ depends on the mass of allele $t$ and the mass and height of the parent allele $t+1$ causing reverse stutter.

$\Pr\left( \bigcup_{t\in\boldsymbol{t}} H_{t}=h_{t} | \bigcup_{a\in\boldsymbol{a}} H_{a}=h_{a},\hat{\boldsymbol{A}}= \hat{\boldsymbol{a}}\boldsymbol{,M=m} \right)$

$$=\prod_{t\in\boldsymbol{t}} Pr(H_{t}=h_{t}\left| M_{t}=m_{t},{M_{t+1}=m}_{t+1},H_{t+1}=h_{t+1} \right).$$

To compute the probability of observing some height $H_{t}$, we use the same reasoning as the one used for $H_{u}$:

| $\Pr{(H}_{t}=h_{t}\left\vert M_{t}=m_{t},{M_{t+1}=m}_{t+1},H_{t+1}=h_{t+1} \right)$ $\boldsymbol{=}p\left( h_{t};\mu_{m_{t}}+h_{t+1}\mu_{m_{t+1}},\sigma_{m_{t}}+{h_{t+1}\sigma}_{m_{t+1}} \right) \left( 1-q_{stut}\left( m_{t+1} \right) \right)+p\left( h_{t};\mu_{m_{t}},\sigma_{m_{t}} \right) \left( q_{stut}\left( m_{t+1} \right) \right)$ | (10) |
| --- | --- |

Equations (1) through (10) enable the calculation of the probability of observing the peak heights in the signal, given the genotype and the mixture proportions of the contributors.
