# Supplementary figures and images for: Four model variants within a continuous forensic DNA mixture interpretation framework: Effects on evidential inference and reporting
Source: PLoS One. 2018 Nov 20;13(11):e0207599. doi: 10.1371/journal.pone.0207599 (PMC6245789; doi:10.1371/journal.pone.0207599)

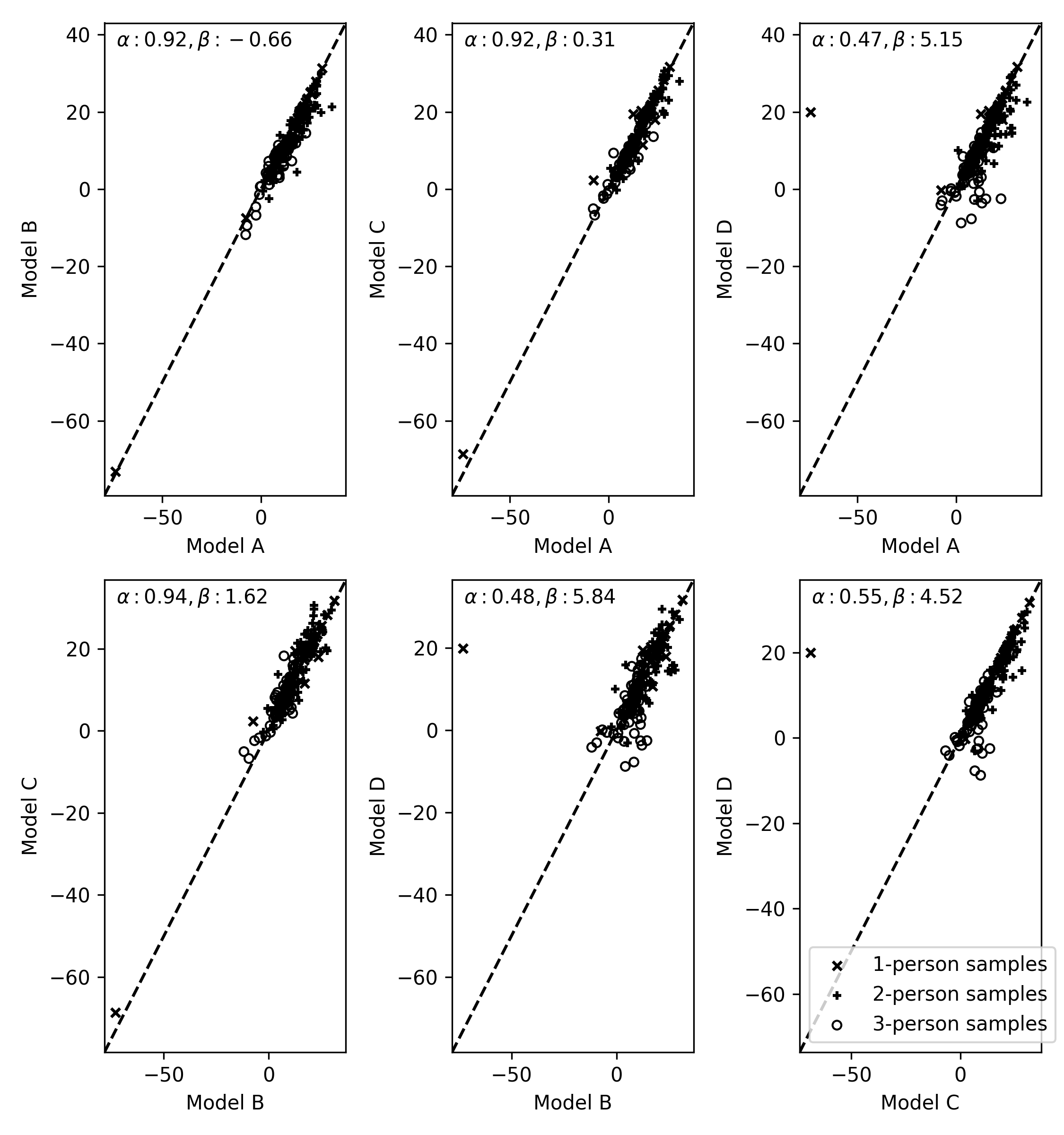

Supplement: S1 Fig — In each plot, the slope α and the intercept β of the best fit linear regression line are shown along with the x = y line. If the LRs do not differ based on the model, the points in the graph would lie along the x = y line and the values for the slope and the intercept would be 1 and 0, respectively. It can be seen from the figure that the slope and intercept of the best fit line for the comparisons of Model A vs Model B (both assume a normal distribution for noise peak heights) and Model C vs Model D (both assume a lognormal distribution for noise peak heights) are closest to the slope and intercept of the x = y line. (TIF) [file pone.0207599.s006.tif]

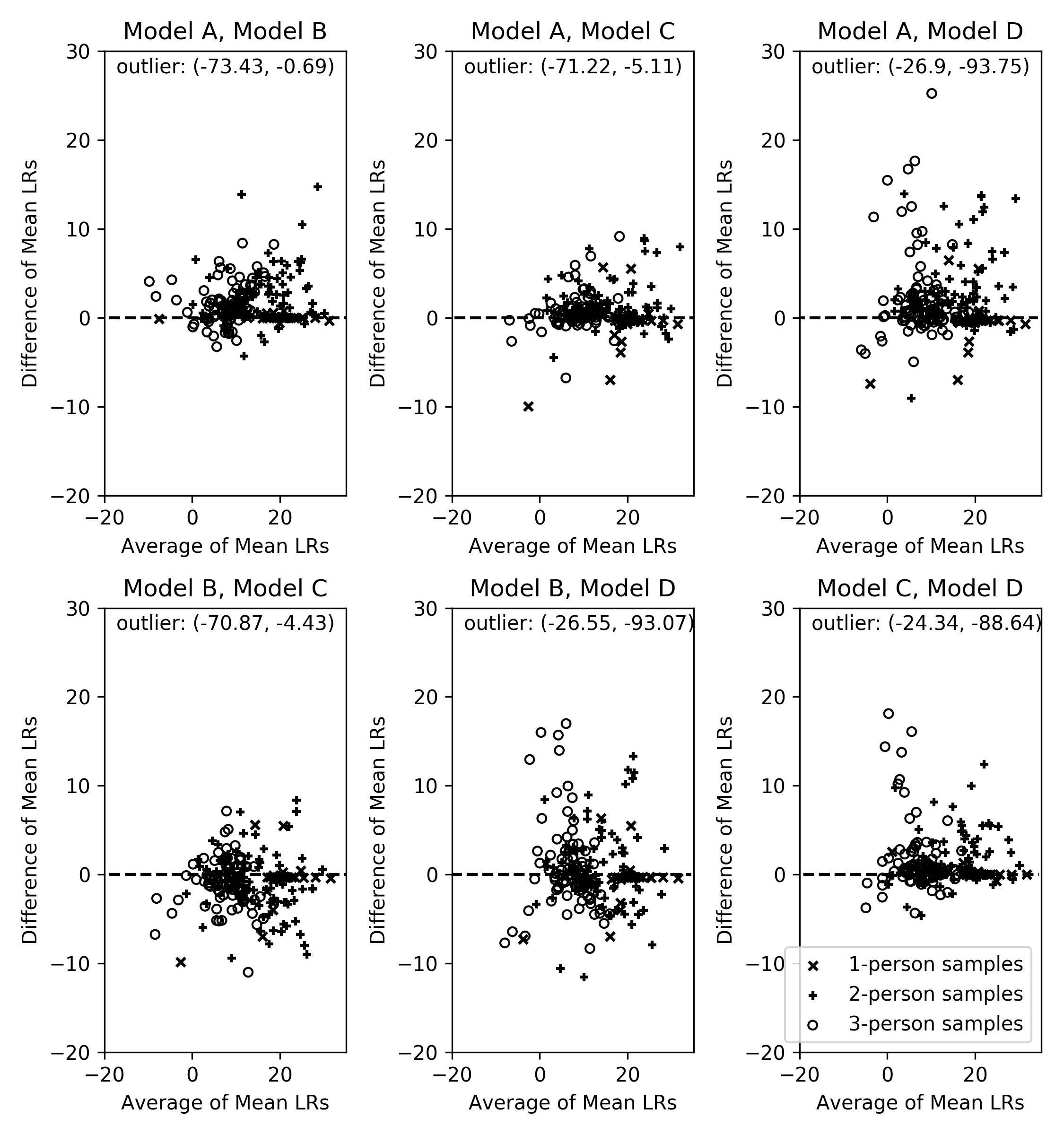

Supplement: S2 Fig — In each plot, the y = 0 line is shown. If the LRs do not differ based on the model, the points in the graph would lie along the y = 0 line. While in most cases, the difference between the mean log10(LR) is small between a pair of models, there are cases where it is large (more than a few orders of magnitude). We also see that, for any given pair of models, there appears to be no dependence of the difference between the mean log10(LR) and its average. There is one large outlier point in each plot that is not shown whose coordinate is reported separately. (TIF) [file pone.0207599.s007.tif]

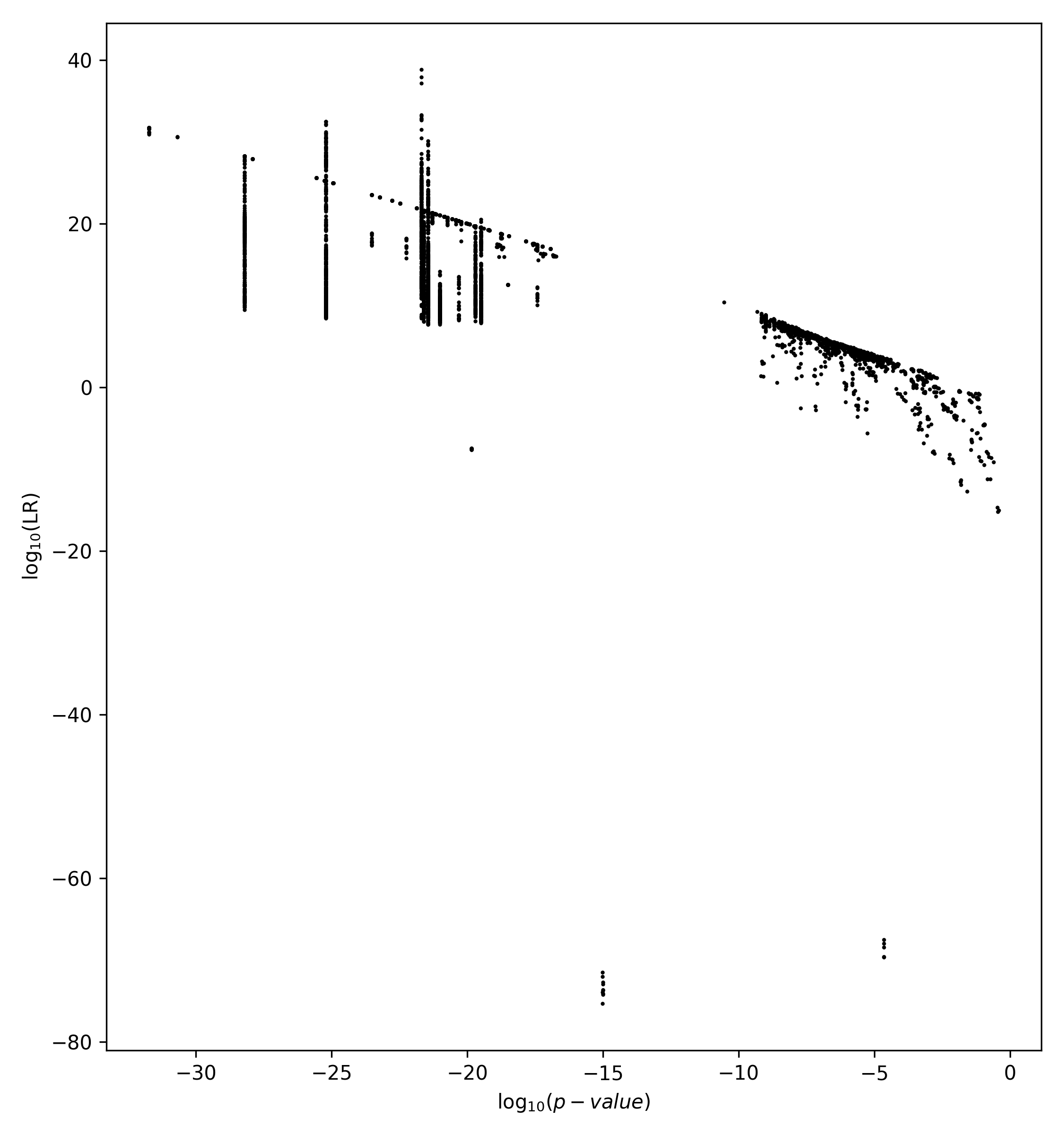

Supplement: S3 Fig — We observe that the p-values decreased with an increase in the LR (Spearman’s rho = -0.75). For p-values greater than 10−9, the p-value is upper bounded by 1/LR as expected. For p-values of 10−9 or lower, the reported value represents only an upper bound to the true p-value. (TIF) [file pone.0207599.s008.tif]
